# Conditional deletion of TRPC1 channel modulates synaptic plasticity, long term depression, and memory extinction in Fragile X syndrome mice

**DOI:** 10.1016/j.isci.2025.113085

**Published:** 2025-07-10

**Authors:** Farah Issa, Xavier Yerna, Thibaud Parpaite, Caren Jabbour, Olivier Schakman, Nicolas Tajeddine, Roberta Gualdani, Philippe Gailly

**Affiliations:** 1 University of Louvain - Institute of Neuroscience - Laboratory of Cell Physiology, 1200 Brussels, Belgium

**Keywords:** Genetics, Molecular biology, Neuroscience

## Abstract

Group I metabotropic glutamate receptors (mGluRs), particularly mGluR5, regulate synaptic plasticity via long-term depression (mGluR-LTD), a process implicated in declarative memory. We previously identified TRPC1, a highly expressed hippocampal ion channel, as a key mGluR5 effector. Using a Cre-tamoxifen system, we acutely deleted *Trpc1* in a Fragile X syndrome (FXS) mouse model, characterized by mGluR5 hyperactivity, enhanced mGluR-LTD, and social deficits. In FXS neurons, mGluR5-evoked currents were elevated and normalized by *Trpc1* deletion. This deletion also improved social behavior and reduced anxiety. Notably, it abolished mGluR-LTD and normalized memory extinction, as shown in behavioral assays. Mechanistically, mGluR5 activation induced ARC protein expression via eEF2K- and ERK1/2-dependent pathways, modulating *Arc* translation and transcription. These findings highlight TRPC1 as a crucial mediator of pathological plasticity in FXS and a potential therapeutic target.

## Highlights

• mGluR5 activates TRPC1 channel, triggering eEF2/ERK1/2 and ARC expression
• mGluR5-induced currents are higher in FXS vs. WT and drop with *Trpc1* gene deletion
• *Trpc1* gene deletion inhibits mGluR5-induced LTD
• *Trpc1* gene deletion corrects the exaggeratedly rapid memory extinction observed in FXS

## Graphical abstract

**Figure:**
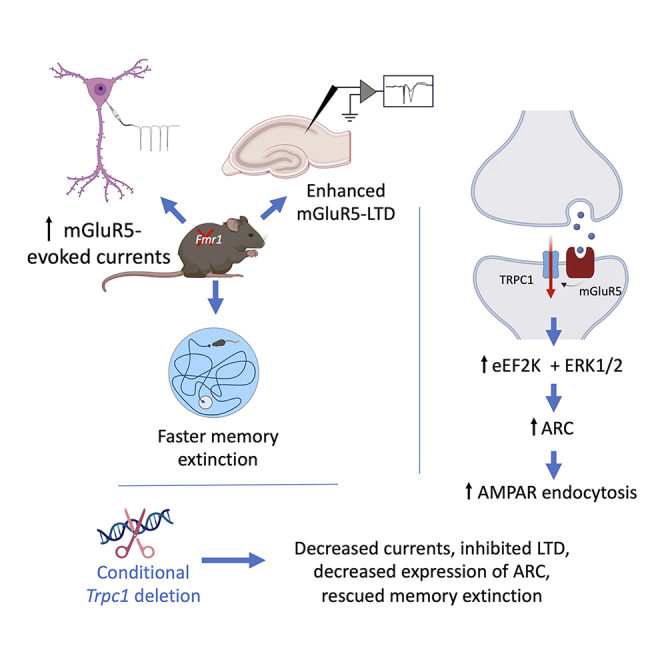

## Introduction

Fragile X Syndrome (FXS) is the most common inherited cause of intellectual disability and autism spectrum disorder (reviewed in^1^). Its symptoms include neurodevelopmental delay, hyperactivity, autistic like behavior, anxiety, and cognitive impairments (reviewed in^2^^,^^3^). It is caused by a mutation in the *Fmr1* gene located on the X chromosome. This gene produces a protein called Fragile X messenger ribonucleoprotein 1 (FMRP), which plays a critical role in the regulation of protein synthesis in neurons. The mutation in *Fmr1* is usually due to the expansion of CGG trinucleotide repeats, leading to gene silencing and a deficiency or complete absence of FMRP. FMRP is an RNA-binding protein that suppresses the translation of multiple mRNAs into proteins at the synapse. It is particularly important in neurons, where local protein synthesis in response to synaptic activity supports synaptic plasticity, learning, and memory.

One of the most important pathways involved in synaptic plasticity that is regulated by FMRP is the metabotropic glutamate receptor 5 (mGluR5) pathway. The activation of mGluR5 by the neurotransmitter glutamate typically leads to increased protein synthesis at the synapse. In the absence of FMRP, the activation of mGluR5 leads to excessive translation of several proteins involved in synaptic plasticity, including the products of immediate-early genes (IEGs) such as the activity-regulated cytoskeleton-associated protein (ARC).^4^^,^^5^ This overactivation of mGluR5 signaling is considered as one of the possible mechanisms implicated in some behavioral symptoms and cognitive impairments of FXS, in particular exaggerated memory extinction, and impaired social interaction and communication (reviewed in^3^^,^^6^). mGluR5, therefore, represents a promising target for the treatment of the disease, and indeed, using murine models of FXS, it has been demonstrated that reducing the expression of mGluR5 by 50% or inhibiting it pharmacologically restores some aspects of normal synaptic plasticity and behavior.^7^^,^^8^^,^^9^ Clinical trials, however, with two different mGluR5 inhibitors (basimglurant and mavoglurant) showed no therapeutic benefit in patients with FXS for reasons as yet unclear,^10^ prompting us to investigate further the importance of the mGluR5 signaling pathway in this syndrome.

Group I mGluRs, notably mGluR5, exert critical regulatory roles in enduring forms of hippocampal synaptic plasticity, including long-term potentiation (LTP) and long-term depression (LTD), pivotal processes underlying memory formation and extinction (reviewed in^11^). LTD is a form of synaptic plasticity characterized by the weakening of synaptic strength, which balances synaptic potentiation and is essential for memory flexibility. mGluR5-induced LTD (mGluR-LTD) can be achieved through various means, such as the application of dihydroxyphenylglycine (DHPG),^12^ a group I mGluR agonist, or physiological activation via paired-pulse low-frequency stimulation.^13^ The maintenance of mGluR-LTD typically involves AMPA receptor (AMPAR) endocytosis^14^ and requires protein expression. This process is important for memory extinction, a mechanism by which previously acquired memories are weakened or suppressed when they are no longer relevant, allowing the brain to update or replace old information with new experiences. In *Fmr1* knock-out mice, the overactivation of the mGluR5 signaling pathway and its consequences on protein translation induce an exaggerated mGluR-LTD^15^ and an accelerated memory extinction^7^^,^^16^

Type I metabotropic glutamate receptors (mGluRs), mGluR1 and mGluR5, are mainly coupled to PLC via G_q/11_ G proteins.^17^^,^^18^ In neurons, we and others reported that they also activate TRPC1, a non-selective cationic channel of the Transient Receptor Potential (TRP) family (canonical subfamily, member1) , which induces an entry of Ca^2+^ and Na^+^, depolarizes the cell, and increases its excitability.^19^^,^^20^^,^^21^

In the present article, we therefore investigated the extent to which the mGluR5-TRPC1 pathway was involved in LTD and related behaviors in FXS. We used a Cre-tamoxifen conditional system to induce an acute deletion of the *Trpc1* gene in a murine model exhibiting FXS and show that this deletion inhibits overactivated mGluR5 signaling pathways, reduces mGluR-LTD, and rescues related behavioral deficits.

## Results

### Metabotropic glutamate receptor 5-induced inward current in fragile X syndrome neurons

FXS mice are characterized by the lack of FMRP protein (Figures S1A and S1B). The metabotropic glutamate receptor theory of FXS postulates that in the absence of FMRP, enhanced signaling though G-protein coupled group I metabotropic glutamate receptors in the brain contributes to many of the abnormalities observed in the disorder.^22^ As we showed previously that mGluR5 specifically activates TRPC1 ion channels, we measured whether this signaling pathway was modified in FXS. Hippocampal neurons were isolated from newborn (P0/P1) mice and cultured 13 days. Cells were voltage-clamped at −60 mV. To prevent neuronal activity, experiments were performed in the presence of 1 μM TTX, 10 μM CNQX, 10 μM D-AP5, 30 μM picrotoxin, and 10 μM bicuculline to inhibit Na^+^ voltage-dependent channels, AMPA, NMDA, and GABA receptors, respectively. To prevent the activation of store-dependent entry of Ca^2+^, the PLC inhibitor U-73122 (5 μM) was also added in the extracellular medium. In these conditions, the stimulation of the FXS neurons with 100 μM DHPG induced an inward current that was significantly bigger than that observed in WT (*p* = 0.023, Figures S1D and S1E). We therefore also measured the expression of TRPC1 which was not modified (Figure S1C), suggesting that FXS indeed presents an exaggerated signaling through mGluR5.

### Generation of a new mouse model characterized by the conditional deletion of TRPC1 in a fragile X syndrome mouse model

**Figure 1:**
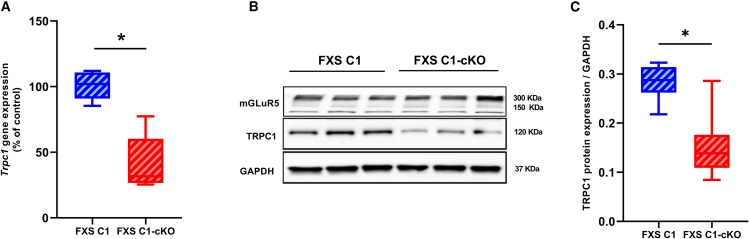
Generation and characterization of FXS C1-cKO mice (A) Trpc1 gene expression measured by RT-qPCR analysis on whole hippocampus of FXS C1 and FXS C1-cKO 10 days post tamoxifen injection, showing a decrease in Trpc1 gene expression in the FXS C1-cKO compared to their control FXS C1. ΔCT of each sample was normalized to Gapdh. ΔCT were then normalized to FXS C1 as control and expressed as percentage (*n* = 7 per group, each n represents 1 hippocampal tissue collected from 1 mouse, Mann-Whitney t-test for nonparametric distribution). (B) Representation of western blot for mGluR5 and TRPC1 proteins measured in whole hippocampal tissues, showing a decrease in TRPC1 protein expression in FXS C1-cKO. (C) Quantitative western blot of TRPC1 protein, reveals a decrease in TRPC1 protein expression in FXS C1-cKO compared to FXS C1 (*n* = 6 per group, each n represents 1 hippocampal tissue collected from 1 mouse, Mann-Whitney t-test). ∗*p* < 0.05.

Fragile X syndrome (FXS) mice lacking FMRP protein^23^ were crossed with transgenic mice *Trpc1* (mice expressing the CreERT2 fusion protein under the control of *CaMKIIα* promotor^20^ to generate a new mouse model of *Fmr1 X*^*-*^*Y Trpc1*^*lox/-*^ *CaMKIICre*^*+/−*^ (herein referred to as FXS C1-cKO) and their littermate *Fmr1 X*^*-*^*Y Trpc1*^*lox/-*^ (here in referred to as FXS C1) used as control (Figures 1 and S2). These mice were injected intraperitoneally for 5 consecutive days with 2 mg of tamoxifen each day, and experiments were performed 10 days after the last tamoxifen injection day. qPCR on hippocampal samples revealed a decrease in *Trpc1* gene expression by 70.3% in FXS C1-cKO compared to their control littermates FXS C1 (FXS C1: mean = 100% ± 3.8, FXS C1-cKO: mean = 29.7% ± 7.7, *p* = 0.02) (Figure 1A). TRPC1 is not only expressed in excitatory neurons but also in interneurons^24^ and in astrocytes.^25^ As *CamKIIα* is only expressed in some excitatory neurons, we therefore expected a reduction in TRPC1 expression in FXS C1-cKO instead of a total loss of the protein. Western blot analysis demonstrated a decrease in TRPC1 protein expression in the hippocampus of FXS C1-cKO compared to FXS C1 (Figures 1B and 1C) (FXS C1: mean = 0.28 ± 0.01, FXS C1-cKO: mean = 0.15 ± 0.02, *p* = 0.01). These results demonstrate an acute conditional deletion of TRPC1 in an adult FXS mouse model. We then compared DHPG-induced currents in cultured neuron from FXS C1 and FXS C1-cKO mice (all treated one week with 2 mM OH-tamoxifen), and observed a significant reduction of these currents in FXS C1-cKO vs. FXS C1 (Figure S1F).

It is well known that TRPC1 is expressed at the plasma membrane forming essentially heterotetramers with TRPC4 and TRPC5. Kollewe and colleagues showed that in TRPC1 knock out brain, the expression of TRPC4 and TRPC5 is decreased by 56% and 67% respectively.^26^ Here, after an acute knock down of TRPC1 in adult mice, we observed unchanged mRNA levels of TRPC4 and TRPC5 in the CA1 region of the hippocampus, suggesting the absence of an early adaptation mechanism (Figure S3). This suggests, but does not prove, that TRPC4 and TRPC5 proteins expression levels are unchanged.

### TRPC1 deletion repairs anxiety phenotype and social interaction deficits observed in fragile X syndrome mice

**Figure 2:**
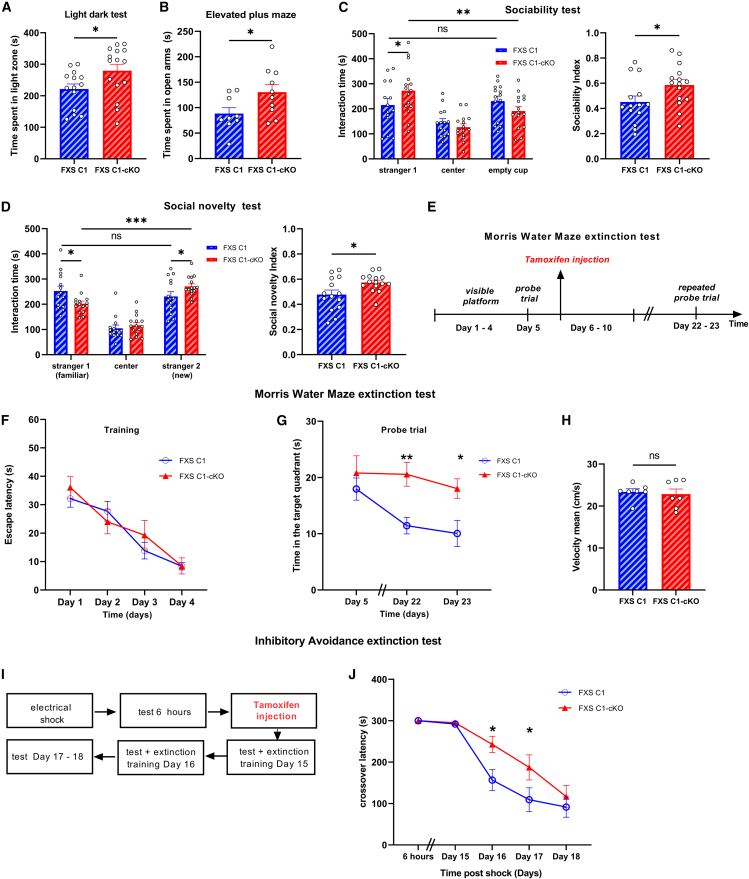
Behavioral evaluation of anxiety, sociability, and memory extinction in FXS C1 and FXS C1-cKO mice (A and B) Light Dark Test (LDT) and Elevated Plus Maze (EPM) assess anxiety behavior in FXS C1 vs. FXS C1-cKO groups. Time spent in the light zone and time spent in open arms were higher in FXS C1-cKO group compared to their control FXS C1 group in LDT and EPM, respectively, showing a lower anxiety level (LDT FXS C1 *n* = 14, FXS C1-cKO *n* = 15, Student’s t test; EPM FXS C1 *n* = 9, C1-cKO *n* = 10, Student’s t test). (C and D) Sociability and Social novelty tests in FXS C1 vs. FXS C1-cKO groups. FXS C1-cKO showed more sociable behavior in both tests than FXS C1 (FXS C1 *n* = 14, FXS C1-cKO *n* = 15, two-way ANOVA). (E) Schematic illustration for Morris Water Maze (MWM) extinction protocol. (F–H) MWM extinction test for FXS C1 vs. FXS C1-cKO groups, showing escape latency (before injecting tamoxifen), time in target quadrant, and velocity mean (on day 5 of the test) respectively, where FXS C1-cKO showed a reduced memory extinction compared to FXS C1 (FXS C1 *n* = 7, FXS C1-cKO *n* = 7, two-way ANOVA for graphs F and G, Student’s t test for graph H). (I) Schematic illustration for Inhibitory Avoidance (IA) extinction protocol. (J) Cross over latency assessed for FXS C1 vs. FXS C1-cKO groups, where FXS C1-cKO showed a reduced memory extinction compared to FXS C1 (FXS C1 *n* = 10, FXS C1-cKO *n* = 10, two-way ANOVA). Values are mean ± SEM. ∗*p* < 0.05, ∗∗*p* < 0.01, ∗∗∗*p* < 0.001, ns not significant.

The behavioral phenotype of FXS mice varies depending on the deletion method of *Fmr1* gene and on the mouse model genetic background.^27^ To check the phenotype developed by the FXS mice strain used in this study, and since the genetic background of FXS and WT littermates differs from that of FXS C1 and FXS C1-cKO, we conducted separate behavioral experiments comparing FXS to their control littermates WT on the one hand (presented in Figure S4), and FXS C1-cKO to their control littermates FXS C1 (both injected with tamoxifen), on the other hand (presented in Figure 2). FXS mice showed an increased anxiety-like behavior when performing both the light-dark test (LDT) and the elevated plus maze (EPM). In those tests, anxiety was assessed by testing the time the mouse spent in the light zone and in the open arms, respectively. FXS mice spent less time in the light zone in LDT (*p* = 0.044 Figure S4A) and in the open arms in EPM (*p* = 0.007, Figure S4B) compared to the WT, indicating an anxiety-like phenotype in those FXS mice. Interestingly, inhibiting the expression of TRPC1 in FXS C1-cKO reduced this anxious phenotype. Indeed, FXS C1-cKO spent more time (FXS C1: mean = 221 ± 16.1, FXS C1-cKO: mean = 279.4 ± 20.9, *p* = 0.038) in the light zone in LDT (Figure 2A) and in the open arms (FXS C1: mean = 88.4 ± 11.4, FXS C1-cKO: mean = 130.5 ± 15, *p* = 0.043) in EPM (Figure 2B) compared to their control FXS C1.

Social interaction deficits are also commonly observed in FXS mouse models.^28^^,^^29^ However, reported results vary, and this is probably partially due to differences in the background of the mice and the protocols used. Here we tested the social behavior of our FXS mice in the three-chamber test and observed that no significant difference was detected in the sociability test (sociability index: *p* = 0.17, Figure S4C). However, contrary to WT mice, FXS presented a reduced attraction to social novelty (social novelty index: *p* = 0.002, Figure S4D). We tested whether knocking down *Trpc1* affected social behavior. FXS C1-cKO mice showed an improved preference (Figure 2C) for interacting with stranger 1 mouse, compared to FXS C1 (sociability index: FXS C1: mean = 0.45 ± 0.04, FXS C1-cKO: mean = 0.58 ± 0.04, *p* = 0.043). FXS C1-cKO mice also spent more time interacting with a new mouse (stranger 2) rather than a familiar one (stranger 1) (Figure 2D), suggesting a more sociable phenotype and an improved social memory post *Trpc1* deletion (social novelty index: FXS C1: mean = 0.47 ± 0.03, FXS C1-cKO: mean = 0.57 ± 0.01, *p* = 0.022). Taken together, these results suggest that the inhibition of TRPC1 expression rescues the anxiety phenotype and the impaired social behavior of FXS mice.

### Acute deletion of TRPC1 rescues the exaggerated memory extinction observed in fragile X syndrome mice

The Morris Water Maze (MWM) assesses spatial reference memory. In a previous publication, we showed that TRPC1 KO mice exhibit normal spatial reference memory but slightly slower learning rates during the early phase of the test.^20^ In the present study, to avoid any potential developmental impairment consecutive to the early age deletion of *Trpc1* gene, FXS C1 and FXS C1-cKO underwent MWM assay, followed subsequently by tamoxifen injection to acutely inhibit TRPC1 expression in neurons of the forebrain. Mice were then readdressed to repeated MWM probe test trials one week after the last tamoxifen injection to test for memory extinction (Figure 2E). Spatial reference memory was also tested for FXS and WT mice, who were then re-addressed again two weeks after the last probe trial, to the repeated probe test trials. Both groups, FXS and WT, presented a similar learning curve (Figure S4F) and normal initial reference memory (probe trial on day 5, *p* = 0.98, Figure S4G) but an enhanced memory extinction for the FXS group, in the repeated probe trials (*p* = 0.003 on day 22), two weeks after the first probe trial (Figure S4G). On the other hand, and as anticipated (mice not yet injected with tamoxifen), the learning process and the initial reference memory were similar for FXS C1 and FXS C1-cKO (Figures 2F and 2G: probe trial on day 5, FXS C1: mean = 17.9 ± 1.9, FXS C1-cKO: mean = 20.8 ± 3.0, *p* = 0.964). However, inhibiting TRPC1 reduced memory extinction seen in FXS mice (Figure 2G), where FXS C1-cKO spent a significantly higher time, in the target quadrant on day 22 (FXS C1: mean = 11.4 ± 1.4, FXS C1-cKO: mean = 20.5 ± 2.1, *p* = 0.004) and 23 (FXS C1: mean = 10.05 ± 2.3, FXS C1-cKO: mean = 18.03 ± 1.7, *p* = 0.017) of the test than FXS C1, suggesting an important role of TRPC1 in memory extinction process. No locomotor impairments were detected in any of the groups (Figures 2H and S4H), suggesting that the observed outcomes were solely dependent on memory processes. Another test to assess memory extinction is the Inhibitory Avoidance (IA) extinction test. This is achieved by measuring the latency for mice to voluntarily enter the dark side of the box (defined by crossover latency) where they have previously received an aversive stimulus (foot shock). Several groups reported a spontaneous decrease in IA memory.^30^^,^^31^ Here, to trigger IA extinction, we gave extinction training on day 15 and 16 of the test consisting of re-exposing the mice to the dark compartment without receiving any foot shock to compete with the initial training memory, and the crossover latency was reassessed (Figure 2I). FXS and WT mice have learned to associate the dark compartment with the foot shock, where they have avoided entering the dark compartment 6 h and 15 days post-shock. FXS group, after being subjected to the extinction training, presented an enhanced memory extinction seen on day 16 of the test (*p* = 0.009, Figure S4E). On the other hand, FXS C1 and FXS C1-cKO presented a similar learning curve (crossover latency measured before extinction procedure and before any injection of tamoxifen), but post tamoxifen injections, and after being exposed to the extinction training, FXS C1-cKO showed a significantly higher crossover latency than FXS C1 on day 16 (FXS C1: mean = 156.6 ± 25.6, FXS C1-cKO: mean = 242.8 ± 19.6, *p* = 0.017) and 17 (FXS C1: mean = 109.2 ± 28.8, FXS C1-cKO: mean = 187.3 ± 30.3, *p* = 0.039) of the test (Figure 2J) implying a significant involvement of TRPC1 in the memory extinction mechanism.

### TRPC1 deletion impairs metabotropic glutamate receptor-induced long-term depression in fragile X syndrome C1-cKO mice

LTD consists of a persistent reduction in excitatory postsynaptic potentials (fEPSP) and is, to some extent, facilitated by endocytosis and decreased surface expression of postsynaptic AMPARs.^32^ The Schaffer collateral (SC)-CA1 pyramidal cell synapse exhibits two forms of LTD^33^: the NMDAR-LTD that can be induced by low frequency stimulation (typically 1Hz for 15 min) and is abolished in the presence of NMDA receptors antagonists^34^^,^^35^ and the mGluR-LTD that can be induced by pair-pulse low-frequency stimulation or by the direct activation of type I mGluR by their agonist, DHPG (100 μM).^12^^,^^36^ mGluR-LTD relies on rapid dendritic protein synthesis^37^ of immediate-early genes (IEG), in particular, activity-regulated cytoskeleton-associated (ARC) protein.^38^ At young ages (30–80 days-old mice), mGluR-LTD is increased in FXS hippocampi compared to WT^37^^,^^39^ but not at later ages.^40^^,^^41^ As first reported by Huber and colleagues,^37^ the most pathognomonic feature of mGluR-LTD in FXS brain slices it that it is maintained even in the presence of the protein synthesis inhibitor anisomycin,^39^^,^^40^^,^^42^ suggesting that elevated levels of synaptic proteins are available to increase the persistence of LTD without *de novo* protein synthesis. Here we assessed the plasticity of SC-CA1 synapses in acute hippocampal slices from adult FXS and WT mice (90–180-day-old) by recording fEPSP in the *stratum radiatum* of the CA1 region in response to SC stimuli. DHPG (100 μM) stimulation resulted in a sustained decrease in fEPSP slope in both FXS and WT brain slices. The plateau levels reached at 60 min post DHPG did not significantly differ between WT and FXS (WT: mean = 76% ± 5, *n* = 14, FXS = 65% ± 7, *n* = 9; not significant). However, as expected, we observed that 20 μM anisomycin (ANISO) almost completely blocked mGluR-LTD in WT brain slices (WT + ANISO = 92% ± 8, *n* = 6; Figures S5A and S5C) but did not have any effect on mGluR-LTD in FXS (FXS + ANISO = 66% ± 5, *n* = 8; Figures S5B and S5C). This was correlated with a significantly higher expression of ARC protein in the hippocampus of FXS compared to WT mice in resting conditions (*p* = 0.028, Figure S5D).

**Figure 3:**
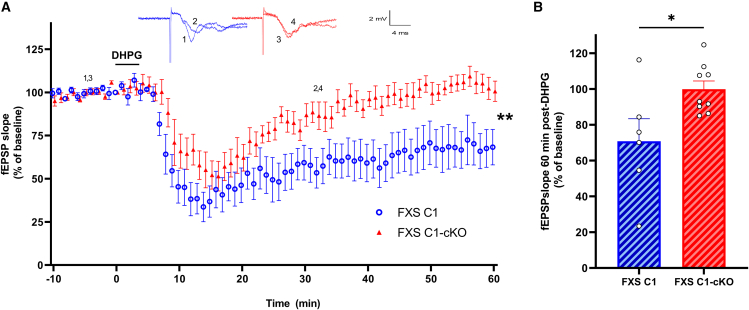
Characterization of DHPG-induced LTD in hippocampal SC-CA1 in FXS C1-cKO mice (A) Time-course of field excitatory postsynaptic potentials (fEPSP) slopes measured in CA1, before and after stimulation with 100 μM DHPG in slices from FXS C1 and FXS C1-cKO. Upper insets show representative traces of fEPSP before and after DHPG. (FXS C1 *n* = 6, FXS C1-cKO *n* = 9, n representing the number of slices from 5 to 7 different mice in each group, two-way ANOVA, F (1, 13) = 9.088, *p* = 0.01, genotype effect). (B) Quantification of the averaged fEPSP slope 60 min post-DHPG induction (FXS C1 *n* = 6, FXS C1-cKO *n* = 9, n representing the number of slices from 5 to 7 different mice in each group, Student’s t test). All experiments were done in the presence of 50 μM picrotoxin and 10 μM D-AP5. Values are means ± SEM. ∗*p* < 0.05, ∗∗*p* < 0.01.

We then assessed the possible involvement of TRPC1 in the process by comparing mGluR-LTD in FXS C1 and FXS C1-cKO mice. In FXS C1 slices, DHPG stimulation induced a sustained decrease in response that did not differ from the one observed in FXS mice (having the same gene defect for *Fmr1* gene but a different genetic background) (Figure 3A). Interestingly, inhibiting TRPC1 expression completely abolished mGluR-LTD response, in FXS C1-cKO, where synaptic strength returned to near baseline levels and was stabilized at around 100% of the pre-stimulation level, 60 min post-DHPG (Figures 3A and 3B) (fEPSP slope 60 min post DHPG: FXS C1: mean = 70.8% ± 12.63, FXS C1-cKO: mean = 99.9% ± 4.58, *p* = 0.026). The correlation between fEPSP slope and stimulus intensity exhibited a comparable pattern in slices from both groups.

### TRPC1 is required for metabotropic glutamate receptor-long-term depression-dependent AMPA receptor endocytosis

**Figure 4:**
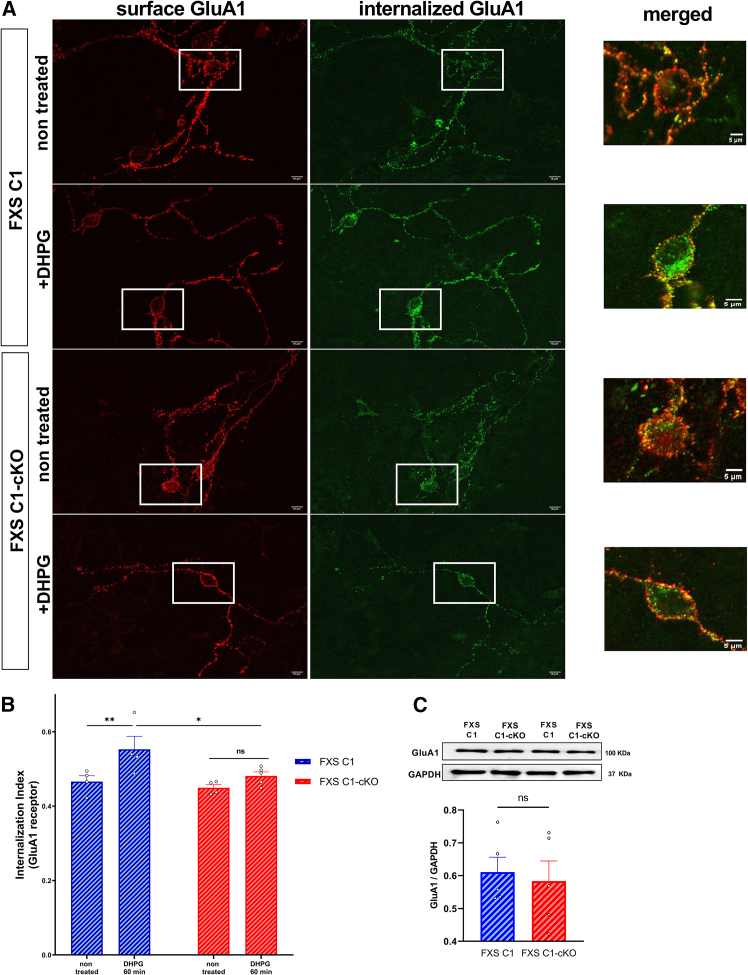
Representative immunostaining of surface and internalized GluA1 subunit of AMPAR in hippocampal neurons (A) Representative immunostaining shows the distribution of surface and internalized GluA1-AMPAR subunit in hippocampal neurons in FXS C1 vs. FXS C1-cKO stimulated or not with 100 μM of DHPG. (B) Quantification of the internalization index (internalized/total) in both groups, showing a significant increase of GluA1 internalization in FXS C1 compared to FXS C1-cKO, 60 min post DHPG stimulation. 10–20 images were collected in each n, (*n* = 4, each n represents one coverslip of hippocampal neuron culture from one mouse), two-way ANOVA, F (1,13) = 5.100, *p* = 0.0418, genotype effect). (C) Representative and quantitative Western blot of samples collected from cultured hippocampal neurons, showing a stable total GluA1 protein expression in both groups (FXS C1 *n* = 5, FXS C1-cKO *n* = 5, each n represents one coverslip of hippocampal neuron culture from one mouse, Student’s t test). All experiments were done in the presence of 50 μM picrotoxin and 10 μM D-AP5. Values are means ± SEM. ∗*p* < 0.05, ∗∗*p* < 0.01, ns not significant.

Given that mGluR-LTD was impaired in FXS C1-cKO, we went further and studied if the endocytosis of AMPAR was affected. Anti-GluA1 subunit (targeting extracellular N-terminal specific domain) was applied to cultured hippocampal neurons, treated or not with 100 μM DHPG, and internalization of GluA1 AMPAR subunit was assessed in the entire neurons 60 min post-DHPG. Treatment with DHPG caused an internalization of the GluA1 subunit in FXS C1. Indeed, the internalized index (internalized/total receptors) presented in Figure 4B shows a significant increase in the internalization of GluA1 only in FXS C1 group 60 min following DHPG treatment (FXS C1 non treated: mean = 0.46 ± 0.01, FXS C1 +DHPG: mean = 0.55 ± 0.03, *p* = 0.009) and not in FXS C1-cKO group (FXS C1-cKO non treated: mean = 0.45 ± 0.01, FXS C1-cKO +DHPG: mean = 0.48 ± 0.01, *p* = 0.25; p (FXS C1+DHPG vs. FXS C1-cKO+ DHPG) = 0.019). Total GluA1 expression was assessed for both groups via western blot, and no difference was detected (FXS C1: mean = 0.61 ± 0.04, FXS C1-cKO: mean = 0.58 ± 0.036, *p* = 0.730) (Figure 4C).

Taken together, these results suggest that TRPC1 plays a critical role in type I mGluR-induced internalization of AMPARs that are responsible for mGluR-LTD.

### TRPC1 is required for metabotropic glutamate receptor-long-term depression-dependent rapid protein synthesis

**Figure 5:**
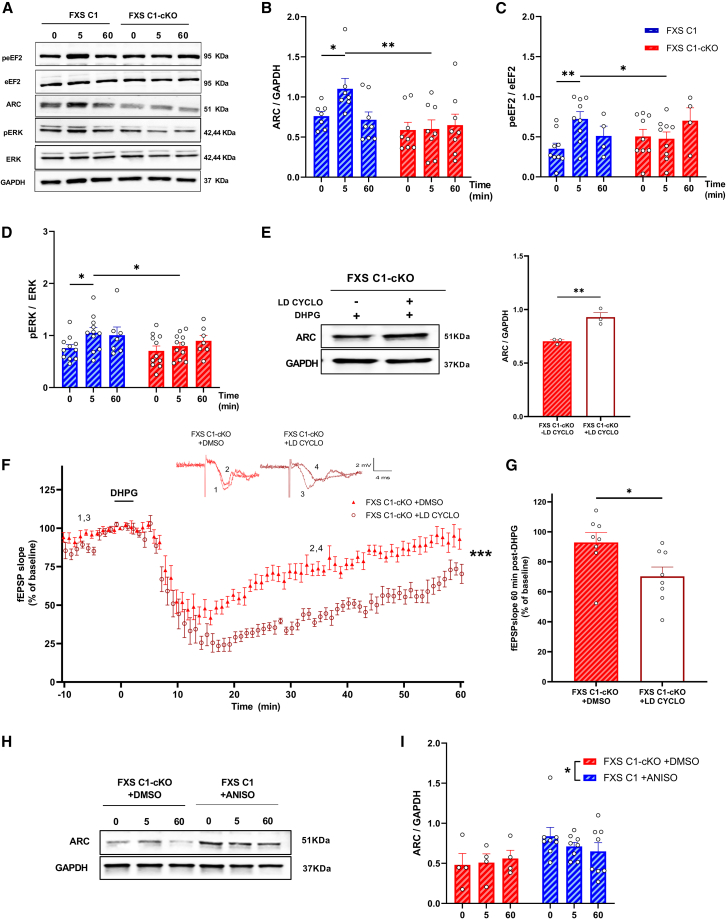
Characterization of hippocampal mGluR-LTD-dependent rapid protein synthesis in FXS C1 vs. FXS C1-cKO mice (A) Representative western blots of protein expression in hippocampal slice*s* stimulated for 0, 5, and 60 min with 100 μM DHPG, revealing an increase in ARC protein expression, and in eEF2 and ERK proteins phosphorylation in FXS C1 but not in FXS C1-cKO in response to DHPG stimulation. (B–D) Quantification of western blots for ARC, peEF2, and pERK proteins in hippocampal slices stimulated with DHPG in FXS C1 and FXS C1-cKO (*n* = 4 to 11 in each group, n representing the number of hippocampal slices from 4 to 8 different mice in each group, two-way ANOVA). (E) Representative and quantitative western blot for ARC protein in hippocampal slices of FXS C1-cKO stimulated with 100 μM DHPG in the presence or absence of low dose of 75 nM cycloheximide (LD CYCLO) 10 min before DHPG, showing an increase in the expression of ARC protein in FXS C1-cKO +LD CYCLO (*n* = 3 per group, n representing the number of hippocampal slices from 3 different mice in each group, Student’s t test). (F) Time-course of fEPSP slopes measured in CA1, before and after stimulation with 100 μM DHPG in FXS C1-cKO slices incubated with 75 nM LD CYCLO or DMSO for 10 min before DHPG. Upper insets show representative traces of fEPSP before and after DHPG. (FXS C1-cKO +DMSO *n* = 8, FXS C1-cKO +LD CYCLO *n* = 9, two-way ANOVA, n representing the number of slices from 5 to 7 different mice in each group, F (1, 14) = 28.49, *p* = 0.0001, genotype effect). (G) Quantification of the averaged fEPSP slope 60 min post-DHPG induction in both groups, showing an abolished LTD in FXS C1-cKO +DMSO but not in FXS C1-cKO + LD CYCLO. (FXS C1-cKO +DMSO *n* = 8, FXS C1-cKO +LD CYCLO *n* = 9, n representing the number of slices from 5 to 7 different mice in each group, Student’s t test). (H and I) Representation and quantification of Western blot for ARC protein in response to DHPG stimulation in FXS C1-cKO +DMSO, and FXS C1 +ANISO treated with 20 μM of anisomycin for 30 min (*n* = 4 to 8 in each group, n representing the number of hippocampal slices from 4 to 6 different mice in each group, two-way ANOVA, *p* = 0.024, genotype effect). All experiments were done in the presence of 50 μM picrotoxin and 10 μM D-AP5. Values are means ± SEM. ∗*p* < 0.05, ∗∗*p* < 0.01 ∗∗∗*p* < 0.001.

Novel protein synthesis is necessary for a maintained mGluR-LTD.^37^ To elucidate the predominant potential pathways engaged by TRPC1 in mediating mGluR-LTD, hippocampal brain slices from FXS C1 and FXS C1-cKO were stimulated with 100 μM DHPG for 0, 5, 60 min, and protein expression or phosphorylation was assessed later-on via western blot analysis (Figure 5A). 5 min after DHPG stimulation, the expression of ARC protein increased significantly in FXS C1 (FXS C1 0 min: mean = 0.76 ± 0.05, FXS C1 5 min: mean = 1.10 ± 0.13, *p* = 0.042) but not in FXS C1-cKO (FXS C1-cKO 0 min: mean = 0.58 ± 0.09, FXS C1-cKO 5 min: mean = 0.60 ± 0.11, *p* = 0.937) where ARC expression persisted at a consistent level over time without noticeable fluctuations (Figure 5B).

In our quest to delineate the factors contributing to triggering ARC expression via TRPC1, we examined two major signaling pathways involved in mGluR-LTD, that of eukaryotic elongation factor 2 kinase (eEF2K), controlling protein translation, and that of extracellular signal-regulated kinases (ERK1/2), controlling both transcription and translation (reviewed in^43^). We observed a rapid phosphorylation of eEF2 protein 5 min after DHPG stimulation in FXS C1 (FXS C1 0 min: mean = 0.35 ± 0.06, FXS C1 5 min: mean = 0.72 ± 0.09, *p* = 0.003) but not in FXS C1-cKO (FXS C1-cKO 0 min: mean = 0.50 ± 0.09, FXS C1-cKO 5 min: mean = 0.47 ± 0.08, *p* = 0.814). (Figure 5C). Similarly, ERK phosphorylation exhibited a significant increase at 5 min in FXS C1 (FXS C1 0 min: mean = 0.75 ± 0.06, FXS C1 5 min: mean = 1.05 ± 0.10, *p* = 0.023) whereas such elevation was not observed in FXS C1-cKO (FXS C1-cKO 0 min: mean = 0.70 ± 0.09, FXS C1-cKO 5 min: mean = 0.79 ± 0.07, *p* = 0.479) (Figure 5D).

### Low dose of cycloheximide rescues impaired metabotropic glutamate receptor-long-term depression in FXS-C1-cKO

Park and colleagues showed that applying a low dose of cycloheximide inhibits general protein synthesis but increases the level of ARC protein.^40^ We cannot rule out the possibility that it may also increase the expression of other IEGs. Here, the application of 75 nM cycloheximide (LD CYCLO) to hippocampal brain slices was indeed capable of increasing ARC expression (FXS C1-cKO -LD CYCLO: mean = 0.70 ± 0.01, FXS C1-cKO +LD CYCLO: mean = 0.92 ± 0.04, *p* = 0.007) (Figure 5E). We, therefore, investigated the possibility of rescuing the impaired mGluR-LTD in FXS C1-cKO by applying a low dose of cycloheximide for 10 min prior to DHPG application in order to increase ARC protein expression. Interestingly, mGluR-LTD was rescued in FXS C1-cKO treated with a low dose of cycloheximide, in which the fEPSP slope has been stabilized at around 70% of the pre-stimulation level, on the opposite to FXS C1-cKO treated with DMSO, which has been stabilized at 92% of the pre-stimulation level (fEPSP slope 60 min post DHPG: FXS C1-cKO +DMSO: mean = 92.93% ± 6.6, FXS C1-cKO +LD CYCLO: mean = 70.31% ± 6.2, *p* = 0.025) (Figures 5F and 5G).

It may appear surprising that the complete inhibition of protein synthesis does not affect mGluR-LTD in FXS mice (see Figure S5), whereas deleting TRPC1 abolishes mGluR-LTD (FXS C1-cKO mice, see Figure 3A), knowing that increasing the expression of ARC protein (and probably other proteins), mimicking the action of peEF2, is sufficient to rescue mGluR-LTD in these FXS C1-cKO mice. We therefore compared the level of expression of ARC in FXS C1-cKO brain slices to FXS C1 treated with 20 μM anisomycin and observed that ARC expression was significantly higher in the latter (Figures 5H and 5I) (*p* = 0.024, genotype effect). This suggests that the level of ARC expression after anisomycin treatment is high enough to allow mGluR-LTD, whereas it is not enough anymore after a more prolonged inhibition of TRPC1 expression (one week after the last tamoxifen injection).

These data show that TRPC1 is required for increasing ARC expression.

### TRPC1 is required for activity-regulated cytoskeleton expression, which is essential for the metabotropic glutamate receptor-long-term depression mechanism

**Figure 6:**
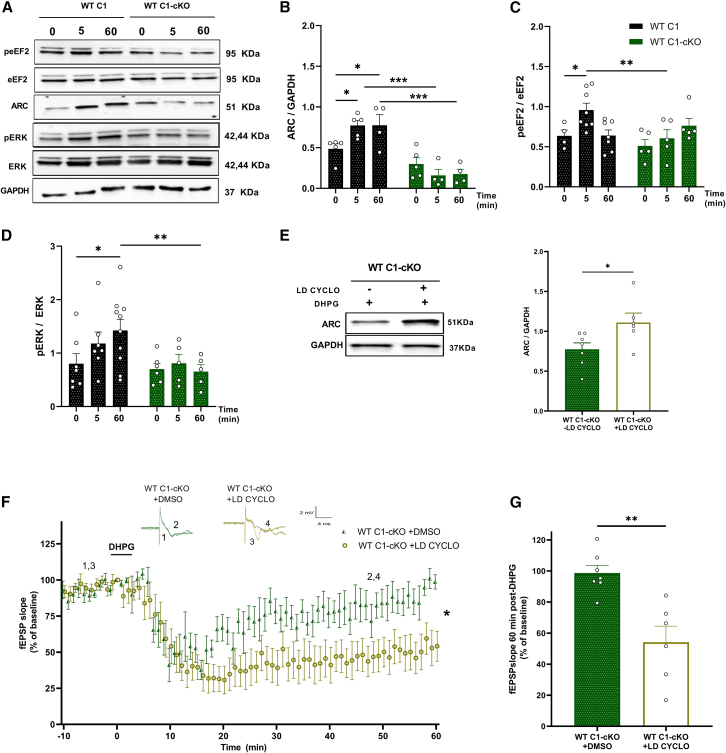
Characterization of hippocampal mGluR-LTD-dependent rapid protein synthesis in WT C1 vs. WT C1-cKO mice (A) Representative western blots of protein expression in hippocampal slices stimulated for 0, 5, and 60 min with 100 μM DHPG revealing an increase in ARC protein expression, and in eEF2 and ERK proteins phosphorylation in WT C1 but not in WT C1-cKO in response to DHPG stimulation. (B–D) Quantification of western blots for ARC, peEF2, and pERK proteins in hippocampal slices stimulated with DHPG in WT C1 and WT C1-cKO (*n* = 4 to 10 in each group, n representing the number of hippocampal slices from 4 to 6 different mice in each group, two-way ANOVA). (E) Representation and quantification of Western blot for ARC protein in hippocampal slices of WT C1-cKO stimulated with 100 μM DHPG in the presence or absence of low dose of 75 nM cycloheximide (LD CYCLO) 10 min before DHPG, showing an increase in the expression of ARC protein in WT C1-cKO +LD CYCLO (*n* = 4 to 8 in each group, n representing the number of hippocampal slices from 3 different mice in each group). (F) Time-course of fEPSP slopes measured in CA1, before and after stimulation with 100 μM DHPG in WT C1-cKO slices incubated with 75 nM LD CYCLO or DMSO for 10 min before DHPG. Upper insets show representative traces of fEPSP before and after DHPG. (WT C1-cKO + DMSO *n* = 7, WT C1-cKO +LD CYCLO *n* = 6, n representing the number of slices from 4 to 5 different mice in each group, two-way ANOVA, F (1, 11) = 5.380, *p* = 0.04, genotype effect). (G) Quantification of the averaged fEPSP slope 60 min post-DHPG induction in both groups, showing an abolished LTD in WT C1-cKO + DMSO but not in WT C1-cKO + LD CYCLO. (WT C1-cKO + DMSO *n* = 7, WT C1-cKO + LD CYCLO *n* = 8, n representing the number of slices from 4 to 5 different mice in each group, Student’s t test). All experiments were done in the presence of 50 μM picrotoxin and 10 μM D-AP5. Values are means ± SEM. ∗*p* < 0.05, ∗∗*p* < 0.01 ∗∗∗*p* < 0.001.

In order to investigate the role of TRPC1 in WT mice, we repeated these measurements and compared WT C1 to WT C1-cKO mice. The effects observed were quite similar to the ones observed in FXS mice. DHPG stimulation significantly increased ARC expression at a 5 min time point in WT C1 (WT C1 0 min: mean = 0.48 ± 0.06, WT C1 5 min: mean = 0.77 ± 0.10, *p* = 0.012) but not in WT C1-cKO (WT C1-cKO 0 min: mean = 0.30 ± 0.07, WT C1-cKO 5 min: mean = 0.15 ± 0.07, *p* = 0.221) (Figures 6A and 6B). In contrast to what was observed in FXS C1 brains, ARC expression remained relatively elevated 60 min after stimulation with DHPG in WT C1 (WT C1 60 min: mean = 0.774 ± 0.13, p (WT C1 0 min vs. WT C1 60 min = 0.017). Note, however, that ARC is globally more expressed in FXS than in WT (Figure S5D). eEf2 was phosphorylated after 5 min stimulation with DHPG in WT C1 (WT C1 0 min: mean = 0.63 ± 0.07, WT C1 5 min: mean = 0.95 ± 0.08, *p* = 0.016) but not in WT C1-cKO mice (WT C1-cKO 0 min: mean = 0.51 ± 0.08, WT C1-cKO 5 min: mean = 0.60 ± 0.10, *p* = 0.477) (Figure 6C) and ERK phosphorylation was also significantly increased upon DHPG stimulation in WT C1 (WT C1 0 min: mean = 0.80 ± 0.18, WT C1 60 min: mean = 1.42 ± 0.20, *p* = 0.017) but not in WT C1-cKO (WT C1-cKO 0 min: mean = 0.69 ± 0.10, WT C1-cKO 60 min: mean = 0.65 ± 0.12, *p* = 0.880) (Figure 6D).

We again investigated the possibility of rescuing the impaired mGluR-LTD in WT C1-cKO by applying a low dose of cycloheximide for 10 min prior to DHPG application in order to increase ARC protein expression (Figure 6E). Interestingly, mGluR-LTD was rescued in WT C1-cKO treated with a low dose of cycloheximide, in which the fEPSP slope has been stabilized at around 54.13% ± 10 of the pre-stimulation level, in contrast to WT C1-cKO treated with DMSO that has been stabilized at 98.65% ± 4.9 of the pre-stimulation level (*p* = 0.001; Figures 6F and 6G). Taken together, these observations show that TRPC1 is involved in ARC upregulation following mGluR-LTD in both FXS and normal conditions.

### Signaling pathways connecting TRPC1 to activity-regulated cytoskeleton protein synthesis

**Figure 7:**
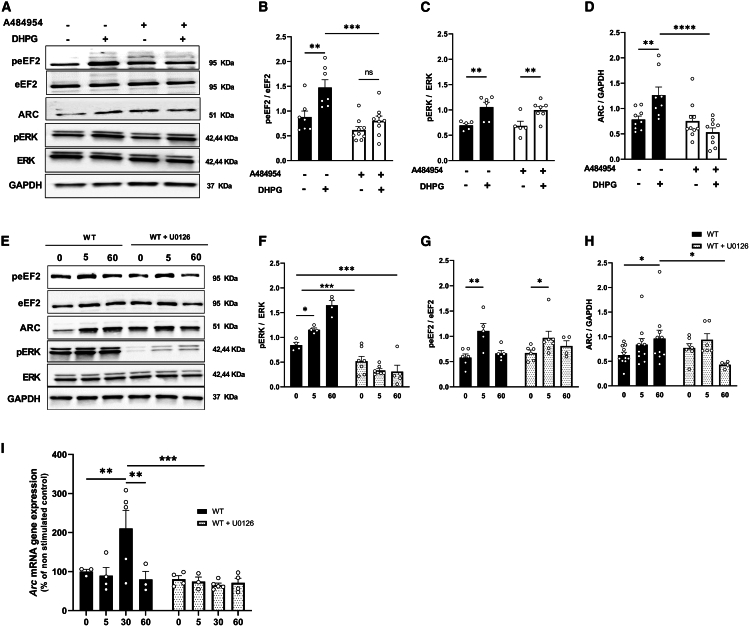
Effects of inhibiting the phosphorylation of eEF2 or ERK on ARC expression in hippocampal WT mice (A–D) Representative and quantitative western blots for peEF2, pERK, and ARC in hippocampal slices stimulated with 100 μM DHPG in WT and WT + A484954, slices were collected 0 min and 5 min after DHPG stimulation (5 μM A484954, eEF2K inhibitor, 20 min before-DHPG, *n* = 5 to 10 in each group, n representing the number of hippocampal slices from 4 to 7 different mice in each group, two-way ANOVA). (E–H) Representative and quantification of western blots for pERK, peEF2, and ARC in hippocampal slices stimulated with 100 μM DHPG in WT and WT + U0126, slices were collected 0, 5, and 60 min after DHPG stimulation (10 μM U0126, MEK-1 and MEK-2 inhibitor, 30 min before-DHPG, *n* = 4 to 11 in each group, n representing the number of hippocampal slices from 4 to 7 different mice in each group, two-way ANOVA). (I) *Arc* gene expression measured by RT-qPCR analysis on WT and WT + U0126, slices were collected 0, 5, 30, and 60 min after DHPG stimulation. ΔCT were normalized to WT at 0 min as control and expressed as percentage (*n* = 3 to 4 in each group, n representing the number of hippocampal slices from 3 to 4 different mice in each group, two-way ANOVA). All experiments were done in the presence of 50 μM picrotoxin and 10 μM D-AP5. Values are means ± SEM. two-way ANOVA ∗*p* < 0.05, ∗∗*p* < 0.01, ∗∗∗*p* < 0.001.

We have shown that the deletion of TRPC1 impairs the phosphorylation and activation of eEF2 and ERK, which control mGluR-induced synthesis of specific proteins such as ARC. We further investigated these pathways but for the sake of simplicity, this was done only on WT brain slices. As TRPC1 activation increases intracellular Ca^2+^ concentration (directly or indirectly, by depolarizing the cell and activating voltage dependent Ca^2+^ channels), we first looked at Ca^2+^- dependent processes. We first tried to objectivate the involvement of calmodulin in the process and used W13, a permeable calmodulin antagonist,^44^ as previously shown by Sethna and colleagues.^45^ We observed that the treatment of brain slices with 70 μM W13 30 min before DHPG stimulation almost abolished mGluR-LTD (Figures S6A and S6B) and completely inhibited mGluR-induced ARC expression (*p* = 0.008) as well as the phosphorylation of eEF2 (*p* = 0.010) and ERK (*p* = 0.005) (Figures S6C–S6F). We then tried to inhibit these pathways using A484954 and U0126, two selective inhibitors of eEF2 kinase and of MEK/ERK, respectively.^46^^,^^47^ As expected, treatment with 5 μM A484954 significantly reduced DHPG-induced eEF2 phosphorylation (at 5 min: WT: mean = 1.47 ± 0.15, WT + A484954: mean = 0.80 ± 0.09, *p* = 0.0002) but did not affect ERK phosphorylation (at 5 min: WT: mean = 1.05 ± 0.09, WT + A484954: mean = 0.99 ± 0.06, *p* = 0.576) (Figures 7A–7C). In this condition, the expression of ARC normally induced by DHPG stimulation was completely inhibited (at 5 min: WT: mean = 1.26 ± 0.16, WT + A484954: mean = 0.53 ± 0.08, *p* < 0.0001) (Figure 7D). Conversely, treatment with 10 μM U0126 largely inhibited ERK phosphorylation (at 5 min: WT: mean = 1.15 ± 0.03, WT + U0126: mean = 0.33 ± 0.03, *p* < 0.0001) but did not affect eEF2 phosphorylation (at 5 min: WT: mean = 1.21 ± 0.1, WT + U0126: mean = 1.01 ± 0.1, *p* = 0.20) (Figures 7E–7G). However, mGluR-induced ARC expression was not affected at 5 min (at 5 min: WT: mean = 0.83 ± 0.1, WT + U0126: mean = 0.93 ± 0.1, *p* = 0.58) but at 60 min post-stimulation (at 60 min: WT: mean = 0.96 ± 0.1, WT + U0126: mean = 0.42 ± 0.03, *p* = 0.013), suggesting that pERK is not necessary in the very early phase of ARC synthesis (translation) but in its late phase (Figure 7H). We therefore investigated its role in *Arc* transcription and observed that indeed, DHPG stimulation significantly increased by a factor of 2 the amount of *Arc* mRNA and that this increase was impaired in slices treated with U0126, suggesting that pERK activates *Arc* transcription (Figure 7I) (*Arc* mRNA gene expression at 30 min: WT: mean = 210% ± 46.3, WT + U0126: mean = 65.7% ± 4.9, *p* = 0.005).

## Discussion

mGluR5-dependent synaptic plasticity plays a crucial role in the reversal learning and in cognitive flexibility, which require suppression of previously acquired memory and acquisition of new information to adapt to novel situations.^45^^,^^48^^,^^49^ Acute inhibition of mGluR5 disrupts behavioral flexibility.^50^ Indeed, the direct chemical stimulation of group I mGluRs with DHPG weakens synapses and induces LTD, which is probably a premise of synapse elimination that is required to refine, consolidate, and update memories. Importantly, the processes of memory extinction and mGluR-LTD have been shown to be slowed down in aging^51^ or in Alzheimer’s disease^52^ and accelerated or exaggerated in Fragile X Syndrome and autism.^22^^,^^53^ mGluR5 therefore represents a therapeutical target for the treatment of such diseases. In fact, what distinguishes mGluR-LTD is its reliance on prompt dendritic protein synthesis,^37^ and intracellular signaling.^54^ Yet, the biochemical pathways connecting mGluR5 activation to protein synthesis and mGluR-LTD have been thoroughly studied but remain somewhat unclear. It is known that mGluR5 activation stimulates a Gαq signaling cascade^55^ and that mGluR-LTD requires intracellular Ca^2+^.^33^ Interestingly, we recently showed that the stimulation of mGluR5 by DHPG activates the TRPC1 channel. This entry that is at least partially independent of endoplasmic reticulum store depletion and persists even after phospholipase C inhibition in neurons.^20^ We therefore investigated whether the mGluR5-TRPC1 pathway was involved in FXS, in particular in the alteration of mGluR-LTD and related behavioral deficits.

TRP channels are widely expressed in mammalian tissues and divided into six subfamilies. Among these subfamilies, TRP canonical (TRPC) channels, encompassing seven members (TRPC1 to TRPC7) are largely expressed across diverse brain regions, such as the hippocampus, amygdala, cerebral cortex, and cerebellum (reviewed in^56^). Recent evidence has underscored their pivotal roles in neuronal development,^57^^,^^58^^,^^59^ neuronal excitability,^60^^,^^61^^,^^62^ and memory processes.^63^^,^^64^ Specifically, TRPC1, highly expressed in the hippocampus and amygdala, has recently emerged as a key player in memory and synaptic plasticity, with its inhibition in mice leading to deficits in working memory, fear conditioning, and synaptic excitability.^20^^,^^65^ TRPC1, showing minimal channel activity when expressed alone, heterotetramerizes with TRPC4 and/or TRPC5 impacting channel function and ion selectivity.^66^^,^^67^^,^^68^ The activation mechanisms of TRPC are diverse, the most known is through G-protein-coupled receptors or receptor tyrosine kinases leading to phospholipase C (PLC) induced formation of diacylglycerol (DAG) and inositol trisphosphate (IP3). The most commonly proposed mechanisms of TRPC1/C4 and TRPC1/C5 activation are the depletion of intracellular stores in cooperation with Orai1 and Stim1 channels^69^^,^^70^^,^^71^^,^^72^ and the direct activation by Gα_q_.^73^ In neurons, we and others reported that the activation of TRPC1 occurs through type I metabotropic glutamate receptors (mGluRs), mGluR1 and mGluR5.^19^^,^^20^^,^^21^ The precise mechanisms by which mGluR5 and TRPC1 cooperate at the synaptic level remain unknown.

**Figure 8:**
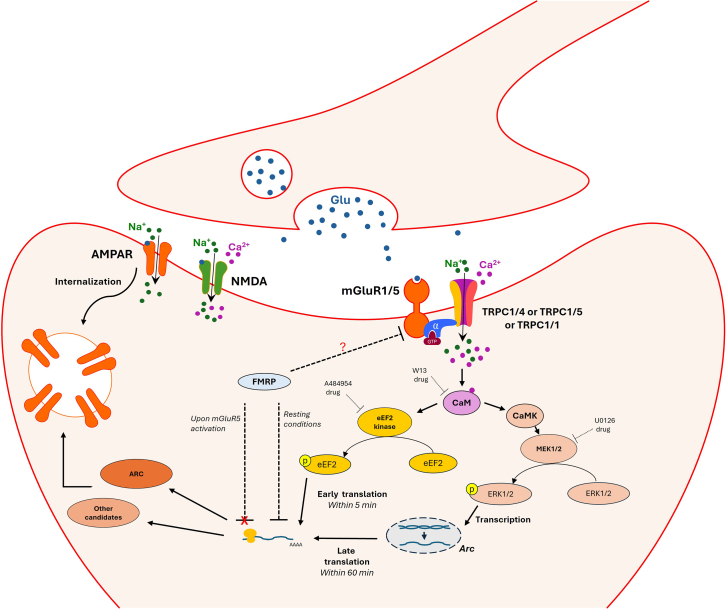
Hypothetical scheme of the involved pathway upon mGluR1/5 and TRPC1 activation in synaptic plasticity The released glutamate activates AMPAR and NMDAR (following sufficient depolarization), along with the perisynaptic mGluR1/5, which, in turn, activates TRPC1/4 or TRPC1/5 heterotetramers or TRPC1 homotetramers. The activation of these channels including TRPC1 further depolarizes the postsynaptic terminal and induces an entry of Na^+^ and Ca^2+^. Ca^2+^ binds to CaM, which in turn activates eEF2 kinase that phosphorylates eEF2. peEF2 inhibits the global elongation but enhances ARC translation and other proteins translation within 5 min. On the other hand, Ca^2+^ entrance activates CaMK that triggers MEK1/2 activation, resulting in ERK phosphorylation. pERK acts on the transcription of many proteins including ARC, resulting in its late translation (within 60 min). ARC, in turn, probably through interacting with the endocytosis machinery leads to the endocytosis of the AMPAR, thus a reduction in the synaptic strength leading to LTD. Under resting conditions, FMRP functions as a translation suppressor, inhibiting the neuronal translation of some proteins, including ARC. Through an unknown mechanism (question mark), it also reduces mGluR5-induced currents. Upon mGluR5 activation, mGluR5 induces FMRP dephosphorylation, leading to FMRP inactivation, thus upregulating the translation of ARC and other proteins. In pathological conditions, such as FXS, FMRP loss leads to increased mGluR5-induced currents and to elevated protein levels resulting in a persistent form of LTD. TRPC1 inhibition results in the loss of eEF2 and ERK1/2 phosphorylation, along with a reduction in ARC protein increase level. This, in turn, reduces some of FXS symptoms, including mGluR-LTD persistence and AMPAR endocytosis. Other candidate proteins and other pathways could be involved.

To investigate the mGluR5-TRPC1 pathway in synaptic plasticity (depicted in Figure 8), we used a mouse model of FXS characterized by an enhanced mGluR5-mediated signaling. These mice presented a typical FXS phenotype characterized by anxiety, autistic-like behavior, increased memory extinction, and persistent LTD. We then constructed an *Fmr1* knockout mouse model in which we were able to acutely inhibit TRPC1 expression in glutamatergic neurons and at a selected period of life using the *CamKIIα Cre-ERT2* tamoxifen system. *CamKIIα* is predominantly expressed in the excitatory neurons of the central nervous system, in particular in the hippocampus, cortex, olfactory bulbs, striatum, and cerebellum, as previously reported.^74^^,^^75^ On the other hand, using a β-galactosidase reporter under the control of the *Trpc1* promoter, we have previously demonstrated that TRPC1 is highly expressed in the hippocampus, particularly within the CA1-CA3 regions and the dentate gyrus. Lower levels of expression were also detected in the cortex, olfactory area, amygdala, and cerebellum.^20^ In our C1-cKO mouse models, TRPC1 is expected to be knocked down in multiple brain regions. We analyzed the synaptic plasticity in the CA1 region of the hippocampus, a structure involved in spatial memory extinction and in fear memory extinction,^49^^,^^76^^,^^77^ but other regions could be involved in other behaviors investigated, in particular the amygdala in anxious phenotype and the prefrontal cortex in social behavior deficits.

Remarkably, the genetic deletion of TRPC1 corrected both exaggerated spatial memory extinction and cognitive extinction impairments detected in FXS mice, as shown by MWM and IA tests, respectively. We did not observe any deficit in the acquisition of reference spatial memory or IA when comparing FXS to WT. This is consistent with previous observations^16^ that demonstrate a normal spatial memory and a normal IA learning but an impaired ability to update existing learning with new information (a new platform location during the MWM reversal task or a change in aversive stimulus in the IA test). Other authors, however, reported impaired spatial learning in FXS rats^78^ (reviewed in^27^).

*ex vivo*, on brain slices, it has been shown that mGluR-LTD in FXS persists even in the presence of a protein synthesis inhibitor.^15^ This is mainly due, according to “the mGluR theory of FXS,”^22^ to the excessive protein synthesis stimulated by mGluR5 activation in the absence of FMRP, a repressor of translation. In *Fmr1* knock-out mice brains, protein synthesis rates are elevated, and there is a higher association of dendritic mRNAs such as PSD-95, ARC, and Map1B with translating polyribosomes compared to wild-type mice.^42^^,^^79^^,^^80^^,^^81^ Among the involved proteins is ARC, an IEG involved in learned behavior. Upon mGluR1/5 stimulation, ARC is swiftly translated in the dendritic spine, where it interacts with the cytoskeleton, allowing AMPAR endocytosis, thus facilitating the late phase and the stability of mGluR-LTD (reviewed in^38^^,^^82^). We show here that the genetic deletion of TRPC1 impairs the induction of ARC translation post mGLuR1/5 stimulation at 5 and 60 min post-DHPG stimulation. This corresponds with the substantial reduction seen in the late phase of LTD, along with the reduction of AMPAR internalization 60 min post-DHPG stimulation. Despite the surprising fact that the complete inhibition of protein synthesis does not affect mGluR-LTD in FXS mice, inhibiting TRPC1 abolishes it. By comparing ARC levels after stimulation, we found significantly higher ARC expression in brain slices treated with anisomycin compared to those observed after prolonged TRPC1 inhibition. This suggests that sufficient ARC expression after anisomycin treatment allows mGluR-LTD, but prolonged TRPC1 inhibition does not maintain these levels. On the other hand, it has been reported that low doses of cycloheximide can increase the synthesis of specific proteins.^83^^,^^84^ According to Park and colleagues,^40^ a low dose of cycloheximide decreases the global elongation, which can potentially enhance the availability of factors required for initiating the translation of specific transcripts (for example, *Arc*) that are usually less actively initiated under normal circumstances, thus mimicking the effect of peEF2. Interestingly, we show that increasing ARC protein expression by applying low dose of cycloheximide rescues the abolished mGluR-LTD in TRPC1 inhibited FXS or WT mice. This comes to a high interest since it highlights that the pathway triggered by mGluR-stimulated TRPC1 exclusively concerns the expression of a few specific IEG, including ARC. However, we observed that increasing the expression of these IEG without stimulating type I mGluRs with DHPG did not trigger LTD. Consequently, we went further and examined the signaling pathways, specifically eEF2 and ERK1/2, which are two kinases known to initiate ARC translation in response to mGluR1/5 stimulation.^40^^,^^45^ mGluR1/5 stimulation induces the phosphorylation of both eEF2 and ERK1/2 in both WT and FXS hippocampal slices, and TRPC1 deletion inhibited it in both mice models. Similar to the effect of TRPC1 knockdown, the application of a calmodulin inhibitor W13 blocked both mGluR5-induced LTD and the increase in ARC protein levels, supporting the hypothesis that mGluR-LTD is likely mediated through TRPC1 and is a calcium-dependent process. Additionally, this inhibition coincided with the suppression of the phosphorylation of both eEF2 and ERK1/2. To gain further insights into the underlying mechanisms, we employed antagonists specific to these kinases in WT hippocampal slices. eEF2 undergoes phosphorylation by eEF2 kinase (eEF2K) that is Ca^2+^/calmodulin dependent, resulting in global translational downregulation but in the increase of the translation of some specific mRNAs such as *Arc*. Inhibiting eEF2K blocked *ARC* translation 5 min post-stimulation. This sparks interest in synaptic plasticity since eEF2 could be considered as a biochemical sensor linking neuronal activity to dendritic spine plasticity,^85^ supporting its role in learning and memory.^86^^,^^87^

ERK1/2 plays an important role in long-term memory formation, along with memory persistence, reconsolidation, and extinction.^88^^,^^89^^,^^90^^,^^91^ DHPG-induced LTD requires ERK1/2 but not p38 activation.^54^ Sethna and colleagues previously showed that the phosphorylation and activation of ERK1/2 induce ARC expression 30 min after the DHPG stimulation of primary hippocampal neurons in culture.^45^ Here, we observed (on hippocampal slices) that reducing ERK1/2 phosphorylation by inhibiting MEK1 and MEK2 does indeed induce a significant reduction of ARC expression at 60 min post-stimulation. However, it did not impact ARC expression at 5 min post mGluR1/5 stimulation, suggesting that early ARC synthesis does not require pERK, whereas its involvement becomes crucial in the later phase. We show that inhibiting the ERK1/2 pathway inhibits *Arc* mRNA formation 30 min post mGluR1/5 stimulation. We therefore conclude that mGluR-LTD involves TRPC1 containing ion channels, the activation of which triggers both peEF2-induced ARC translation and pERK1/2-induced *Arc* transcription (Figure 7). The process plays a crucial role in reversal learning and cognitive flexibility.

It is also noteworthy to mention that in this study, we also show that knocking down TRPC1 resulted in reducing both anxious and impaired social behavior phenotypes seen in FXS mice. It is well known that TRPC1 tends to form heterotetramers with TRPC4 and TRPC5^64^^,^^68^^,^^92^ which are all highly expressed in the hippocampus, the amygdala, and the cortex. When co-expressed with these channels, TRPC1 significantly influences their biophysical properties.^93^TRPC5 KO and TRPC4 KO mice are less anxious when presented with stimuli triggering innate fear responses.^94^ These results, taken together, suggest that TRPC1 exerts its effects, at least in the context of anxiety, through its heteromultimerization with TRPC4 and TRPC5 in different brain regions as mentioned above. However, several studies show that a significant proportion of TRPC1 forms homotetramers that, unlike other members of the TRPC family, reside preferentially within the ER.^92^^,^^95^^,^^96^ Their contribution to the phenotype observed cannot be excluded.

In conclusion, this study suggests that TRPC1 plays a role in reversal learning and adaptability, as well as in other behavioral phenotypes, including anxiety and social interactions. Therefore, TRPC1 may represent a promising therapeutic target for disorders in which these processes are altered, such as FXS.

### Limitations of the study

We investigated synaptic plasticity in the CA1 region of the hippocampus, a brain structure involved in the extinction of spatial and fear memories. However, the molecular mechanisms triggered by mGluR5 activation identified in this region may differ in other brain areas where TRPC1 is also expressed, such as the amygdala, which is implicated in anxiety-related behaviors, and the prefrontal cortex, which is associated with deficits in social behavior.

## Resource availability

### Lead contact

Further information and requests for resources and reagents should be directed to and will be fulfilled by the corresponding author, Philippe Gailly (philippe.gailly@uclouvain.be).

### Materials availability

• This study did not generate new unique reagents.
• Commercially available materials are denoted in the article and the method details.

### Data and code availability

• Data reported in this article will be shared by the lead contact upon reasonable request.
• No unpublished custom code, software, or algorithm was used in this study.
• Any additional information required to reanalyze the data reported in this article is available from the lead contact upon request.

## Acknowledgments

This study was supported by a Concerted Research Action from the General Direction of Scientific Research of the French Community of Belgium, and the Belgian Fund for Scientific Research (FNRS, grant CDR-J.0065.21 and PDR -T.0089.2).

## Author contributions

Conceptualization, F.I. and P.G.; experimentation, F.I., X.Y., T.P., C.J., and O.S.; data analysis, F.I., X.Y., T.P., C.J., O.S., N.T., R.G., and P.G.; writing – original draft, F.I. and P.G.; writing – review and editing, F.I., N.T., R.G., and P.G.; funding acquisition, P.G. All authors approved the final article.

## Declaration of interests

The authors declare no competing interests.

## STAR★Methods

### Key resources table

**Table:** 

REAGENT or RESOURCE	SOURCE	IDENTIFIER
**Primary antibodies**		

Rabbit polyclonal anti-FMRP	Cell Signaling Technology	Cat# 4317, RRID:AB_1903978
Rabbit monoclonal anti-mGluR5	Cell Signaling Technology	Cat# 55920, RRID:AB_2734718
Rabbit monoclonal anti-AMPA Receptor 1 (GluA1)	Cell Signaling Technology	Cat# 13185, RRID:AB_2732897
Rabbit polyclonal anti-eEF2	Cell Signaling Technology	Cat# 2332, RRID:AB_10693546
Rabbit polyclonal anti-peEF2 (Thr56)	Cell Signaling Technology	Cat# 2331, RRID:AB_10015204
Rabbit monoclonal anti-ERK1/2 (p44/42 MAPK)	Cell Signaling Technology	Cat# 4695, RRID:AB_390779
Rabbit recombinant monoclonal anti-phosphoERK1/2 (p44/42 MAPK) (Thr202/Tyr204)	Cell Signaling Technology	Cat# 4370, RRID:AB_2315112
Rabbit recombinant monoclonal anti-GAPDH	Cell Signaling Technology	Cat# 2118, RRID:AB_561053
Rabbit monoclonal anti-TRPC1	Abcam	Cat# ab192031
Rabbit monoclonal anti-ARC	Abcam	Cat# ab183183

**Secondary Antibodies**		

Polyclonal Goat anti-rabbit Immunoglobulins/HRP	Agilent Dako	Cat# P0448
Polyclonal Donkey Alexa Fluor™ 488 anti-rabbit IgG	Thermo Fisher	Cat# A21206
Polyclonal Donkey Alexa Fluor™ 568 anti-rabbit IgG	Thermo Fisher	Cat# 10042
ProLong™ Gold Antifade Mountant with DNA Stain DAPI	Thermo Fisher	Cat# P36931

**Experimental models: strains and cell models**		

Fmr1 KO B6.129P2-*Fmr1*^*tm1Cgr*^/J	The Jackson Laboratory	Strain# 003025 IMSR_JAX:003025
Primary hippocampal cells	This paper	NA

**Oligonucleotides**		

*Trpc1* gene	Eurogentec	*Fw: CAGAAGGACTGTGT* *GGGCAT* *Rv: CAGGTGCCAATGAAC* *GAGTG*
*Trpc4 gene*	Eurogentec	*Fw: AAGCCAAGTGGAGAG* *AAGCA* *Rv: ATCGGAGCTGGAGA* *CACACT*
*Trpc5* gene	Eurogentec	*Fw: GCTGAAGGTGGCAAT* *CAAAT* *Rv: AAGGTTGCTTCTGGGT* *GAGA*
*Arc* gene	Eurogentec	*Fw: CCTGCTCTTACCAGC* *GAGTC* *Rv: CATCCCTTTGGGAGTCAGCC*

**Chemicals, peptides, and compounds**		

(*RS*)-3,5-DHPG	Tocris	Cat. No. 0342
D-AP5	Tocris	Cat. No. 0106
Picrotoxin	Tocris	Cat. No. 1128
Anisomycin	Tocris	Cat. No. 1290
U0126	Tocris	Cat. No. 1144
W-13 hydrochloride	Tocris	Cat. No. 0361
U-73122	Tocris	Cat. No. 1268
Cycloheximide	Tocris	Cat. No. 0970
A-484954	MedChemExpress	Cat. No.: HY-110096
U0126	Sigma Aldrich	CAS No. 109511-58-2
OH-tamoxifen	Sigma Aldrich	CAS No. 68047-06-3
Tamoxifen	Sigma Aldrich	CAS No. T5648
TTX	Sigma Aldrich	CAS No. 554412
Bicuculline	Sigma Aldrich	CAS No.485-49-4
CNQX	Sigma Aldrich	CAS No.115066-14-3
Poly-L-lysine	Sigma Aldrich	CAS No. P2658
Neurobasal™ Medium	Thermo Fisher	Cat# 21103049
penicillin-streptomycin	Thermo Fisher	Cat# 15070063
B27 Supplement	Thermo Fisher	Cat# A3582801
GlutaMAX™ Supplement	Thermo Fisher	Cat# 35050061
Fetal Bovine Serum (FBS)	Thermo Fisher	Cat# A5670701
Pierce™ BCA Protein Assay Kits	Thermo Fisher	Cat# 23227
iTaq™ Universal SYBR® Green One-Step Kit	BioRad	Cat# #1725150

**Software and algorithms**		

Prism	GraphPad	RRID: SCR_002798
Fiji	ImageJ	RRID: SCR_002285
EthoVision 6.1, Noldus	Noldus	RRID:SCR_000441
Scientific illustration (graphical abstract)	BioRender	https://BioRender.com
WinLTP 2.20 M,X-Series	WinLTP	see ref. ^97^

### Experimental model and study participant details

#### Ethical approval

All animals were housed and handled according to the Belgian Council on Animal Care guidelines based on protocols approved by the Animal Ethics Committee of the Université catholique de Louvain (2021/UCL/MD/020). The present study was performed on male 3–6-month-old mice. Animals were given access to food and water *ad libitum*. At appropriate experimental time points, all animals were euthanized through decapitation.

#### FXS C1 conditional knockout mice

FXS mice are *Fmr1* knockout mice (*Fmr1 X*^*-*^*X*^*-*^ and *Fmr1 X*^*-*^*Y*). These mice were obtained from The Jackson Laboratory (strain #003025) where exon 5 of the *Fmr1* gene was replaced by a neomycin resistance cassette resulting in a defection in the *Fmr1* gene (Figure S1). These mice were intercrossed to obtain a homozygous colony of B6.129P2-Fmr1^tm1Cgr/J^ background. Additionally, they were crossed with WT C57BL/6J mice to produce littermates with WT and FXS genotypes (same genetic background, Jackson strain #000664; Figure S2C).

The generation of *Trpc1*^−/−^and *Trpc1*^*lox/-*^ *CaMKII Cre ERT2* mice has been described previously^20^^,^^65^ (Figures S2A and S2B). Briefly, mice were built by having the second exon of *Trpc1* gene flanked with loxP site. These mice were bred with PGK-Cre recombinase mice line to obtain a constitutive *Trpc1* knockout mouse line. Heterozygous mice were further bred to obtain homozygous mice on a mixed genetic C57BL6/129S1/Sv background. The *Trpc1*^*lox/lox*^ were then crossed with mice expressing the CreERT2 fusion protein under the control of the regulatory elements of the *CaMKIIα* (calcium/calmodulin-dependent protein kinase II alpha) promoter.^74^^,^^75^^,^^98^ The FXS mice were crossed with transgenic mice *Trpc1*^*lox/lox*^ *CaMKIICre-ERT2 and* knockout mice *Trpc1*^−/−^ to generate both groups FXS *Trpc1*^*lox/lox*^ *CaMKIICre-ERT2* and FXS *Trpc1*^-/-^. Littermates were further interbred to obtain homozygous mice on a mixed genetic background of FXS *Trpc1*^*lox/lox*^ *CaMKIICre-ERT2* and FXS *Trpc1*^*-/-*^. These mice were then bred together to generate littermates with *Fmr1 X*^*-*^*Y Trpc1*^*lox/-*^ (stated here as FXS C1) and *Fmr1 X*^*-*^*Y Trpc1*^*lox/-*^ *CamKIICre-ERT2*^*+/-*^ (stated here as FXS C1-cKO) (Figure S2D). Genomic DNA was extracted from the tail using HotSHOT solution and PCR genotyping was performed to identify the genotype of each mouse littermate. Identified male FXS C1 and FXS C1-cKO were both injected intraperitoneally with 100 μL of a solution containing 20 mg/ml of tamoxifen (diluted in sunflower oil and 100% ethanol) per day for five consecutive days and experiments were conducted during the 2nd and 3rd week post-tamoxifen-treatment. No injections were administered to the FXS and WT mice. When the role of TRPC1 was studied in WT mice, we compared mice having two *lox Trpc1* alleles expressing or not the Cre recombinase (here below referred to as WT C1 vs WT C1-cKO) both injected with 20 mg/ml tamoxifen. In this study, only male mice were used for all experiments.

### Method details

#### RNA extraction and Real time qPCR

Total RNA was purified using Trizol method and RNA expression was evaluated in the whole extracted hippocampus for *Trpc1*, in the CA1 region of the hippocampus (CA1 region was cut out from hippocampal slices sections) for *Trpc1*, *Trpc4*, and *Trpc5,* and on stimulated hippocampal slices for *Arc*. RT-qPCR primers were as the following: *Trpc1:* Fw: CAGAAGGACTGTGTGGGCAT, Rv: CAGGTGCCAATGAACGAGTG, *Trpc4*: Fw: AAGCCAAGTGGAGAGAAGCA Rv: ATCGGAGCTGGAGACACACT, *Trpc5*: Fw: GCTGAAGGTGGCAATCAAAT Rv: AAGGTTGCTTCTGGGTGAGA, and *Arc*: Fw: CCTGCTCTTACCAGCGAGTC, Rv: CATCCCTTTGGGAGTCAGCC. RT-qPCR was performed using 5 μL of cDNA, 12.5 μL of SyberGreen Mix (BioRad, Temse, Belgium), and 250 μM of each primer in a total reaction volume of 25 μL. The reaction was initiated at 95°C for 3 min and was followed by 40 cycles of denaturation at 95°C for 10 s, annealing at 60°C for 1 min, and extension at 72°C for 10 s. The data were recorded on a DNA Engine Opticon RT-qPCR Detection System (BioRad), and the cycle threshold (Ct) values for each reaction were determined using analytical software from the same manufacturer. To normalize the data, *Gapdh* gene was used as a housekeeping gene.

#### Western Blot analysis

Whole hippocampi or stimulated slices hippocampal sections of 350 μm-thick (see below) were taken and snapped frozen in liquid nitrogen. Proteins were extracted using RIPA buffer (25 mM Tris HCl pH 7.6, 150 mM NaCl, 1% NP-40, 1% sodium deoxycholate, and 0.1% SDS) for 2 h at 4°C. The samples were clarified by centrifugation at 10,000 g for 5 min and supernatant was collected. The protein concentration was determined by bicinchoninic acid protein assay kit (BCA, Pierce) and the absorbance was subsequently measured with NanoDrop spectrophotometer. Samples were heated for 10 min at 70°C. 25 μg of protein per sample was loaded on a 10% SDS-polyacrylamide gel (Biorad) and transferred to a nitrocellulose membrane. Cut membranes were then blocked with either 5% of non-fat milk or 5% of BSA, and were incubated overnight in appropriate antibodies anti-FMRP (1/1000, BSA, Cell Signaling #4317), anti-mGluR5 (1/1000, milk, Cell Signaling) #55920), anti-TRPC1 (1/1000, milk, Abcam #ab192031), anti-GluA1 (1/1000,milk, Cell Signaling #13185), anti-eEF2 and anti-peEF2 (1/1000, BSA, Cell Signaling #2332 and #2331 respectively), anti-ARC (1/1000, milk, Abcam #ab183183), anti-ERK1/2 and anti-pERK1/2 (1/20000, BSA, Cell Signaling #4695 and #4370 respectively), anti-GAPDH (1/20000, milk, Cell Signaling #2118). On the second day, membranes were incubated with secondary antibodies anti-rabbit coupled to peroxidase (Dako/P448) for 1 hour at room temperature and peroxidase was detected with using SuperSignalTM West Pico PLUS Chemiluminescent Substrate (ThermoFisher). Membranes were imaged using Fusion Pulse platform (Vilber) and analyzed in Fiji (ImageJ) where values were normalized to the GAPDH protein.

#### Behavorial assays

Littermates male mice, aged 3 to 6 months, were used for behavioral testing. The FXS and their controls (WT) were on a C57BL/6J background (Jackson Laboratory: RRID:IMSR_JAX:000664), while the FXS C1-cKO and their FXS C1 controls were on a mixed genetic background. Mice were subjected to either anxiety and sociability tests, Morris Water Maze (MWM) extinction test, or Inhibitory avoidance (IA) extinction test, before being sacrificed to western blot or electrophysiological analysis.

##### Light dark test

The light dark test (LDT) was used to assess unconditioned anxiety-like-behavior in mice. LDT consists of a box divided into two sections of equal size by a partition with a door, with one side illuminated while the other side remained dark. Mice were first placed into the dark half of the box and were allowed to move freely between the two chambers for 10 min. Time spent in the illuminated chamber was recorded by a video tracking system (Ethovision 6.1) for a period of 10 min.

##### Elevated plus maze

The elevated plus maze (EPM) was performed as described previously.^99^ Briefly, animals were placed on the center intersection of a 4-arm radial plus maze consisting of two opposing open arms (exposed area) and two opposing closed arms (safe area). The time spent in each arm was recorded by a video tracking system (Ethovision 6.1) for a period of 5 min.

##### Three-chamber social test

The three-chamber social test was used to assess sociability and interest in social novelty. It was performed as previously described.^100^ Briefly, the apparatus consisted of three-chambered box where the walls to the center chamber had in-cutout doors allowing movement between chambers. The two side chambers contained small wire cages, in order to later house stranger mice. The test consisted of three 10 min sessions (habituation, sociability, and social novelty). First the test mouse was placed in the center chamber and was habituated to the empty box for 10 min. Then, to test the sociability, a gender/age-matched stranger was placed into either of the side chambers under the wire cage and the other chamber remains empty. The test mouse was allowed, again, to freely explore between chambers for 10 min. At the end, to test the social novelty, a second mouse (new mouse) was placed into the other side chamber and the test mouse was allowed to move freely within the apparatus for another 10 min. The entire apparatus was wiped with 70% ethanol between mice to eliminate odor cues between animals. The time spent interacting with each mouse or empty cage (nose ≤ 2cm) was recorded by a video tracking system (Ethovision 6.1) for a period of 10 min in each test. Results are expressed as a sociability index (time spent with stranger 1 divided by (time spent with stranger 1 + time spent in empty chamber)) and a social novelty index (time spent with stranger 2 divided by (time spent with stranger 1 + time spent with stranger 2).

##### Morris Water Maze extinction test

The Morris Water Maze (MWM) was used to assess spatial learning and memory.^101^ Tests were conducted in a circular black tank, 113 cm in diameter, divided into four quadrants. The tank was filled with water (26°C) and several visual cues were placed around the pool. A circular platform (8 cm in diameter) was placed 2 cm beneath the water level in a well-defined specific quadrant. During the spatial learning phase, the mice were given a trial per day to learn to locate the visible platform in the target quadrant. If they are not able to find the platform during a period of 1 min, they are placed on top of the platform. During the probe test, on day 5 of the test, the platform was removed from the quadrant and the mice were introduced again to the MWM to test their memory. The maximum trial was 60 s and the time latency to reach the platform, the swim speed, and the time spent in each quadrant were measured by a video tracking system (Ethovision 6.1). In order to measure spatial memory extinction, FXS C1 and FXS C1-cKO mice were then injected with tamoxifen, and 1-week post the last tamoxifen treatment, probe test trials were repeated by placing the mice for 1 min in the water maze from which the platform had been already removed. FXS and WT groups did not receive tamoxifen injection, but they were also subjected to repeated probe tests two weeks after their first probe test.

##### Inhibitory avoidance extinction test

The inhibitory avoidance (IA) test was performed as previously described^102^ with minor modifications. Briefly, the arena consisted of a square box (25 × 25 × 25 cm) containing an electrifiable grid floor placed on a pressure plate. The box was divided into two illuminated (safe) and dark (shock side) compartments, separated by a trap door. On the first day of the test, the mice were habituated for 1 min in the light compartment. Once the door opened, the mice were allowed to enter the dark compartment. After stepping completely into the dark compartment with all four paws, the door was closed and the mice received a single scrambled foot-shock (0.5mA, 2.0 sec) through the electrical grid. The mice, post-shock, were kept for 30 s in the dark compartment before being removed safely to their home cage. 6 hours post-shock, the mice were reintroduced again to the light compartment where the door was open, and the crossover latency, defined as time spent in the light compartment before entering the dark compartment, was assessed. Then, to measure memory extinction, FXS C1 and FXS C1-cKO mice were injected with tamoxifen for 5 consecutive days. 1-week post the last tamoxifen treatment, mice were reintroduced to the light compartment and the crossover latency was reassessed. On day 15 and 16 of the test, once the mice entered the dark compartment, they were subjected to an extinction training where mice were allowed to explore the dark compartment of the box for 200 s in the absence of foot-shock. In case of avoiding completely the dark compartment, mice were introduced manually to the dark compartment. The crossover latency was assessed from day 15 till 18. FXS and WT groups did not receive tamoxifen injection, but they were also subjected to the extinction training and the cross-over latency assessment over consecutive days.

#### Primary hippocampal cell culture

Coverslips were coated with poly-D-lysine for 3 hours in 24 well plates, in incubator at 37°C, and then washed three times with PBS before plating the neurons (two hippocampi from the same pup/coverslip). Mouse pups (0-1P) were sacrificed, and the hippocampi was carefully removed and placed into ice-cold PBS solution. The hippocampal tissue was then dissociated with a sterilized glass pipette, and the tube was left undisturbed for 2 min to allow cell debris to settle down. The cell supernatant was transferred into a new tube of ice-cold FBS and was centrifuged for 5 min, at 4°C, 1000 RPM. the pelleted cells were resuspended in 1 ml of warm cell neuronal culture medium (Neurobasal medium supplemented with glutamine, B27 and penicillin-streptomycin). Cells were plated on coverslips and 2 hours later, the medium was replaced by fresh one. After 1 week, 2 mM OH-tamoxifen (diluted in 100% ethanol) was added daily to the cell culture (for both groups FXS C1 and FXS C1-cKO) over 1 week. Cells were maintained at 37°C in a humidified atmosphere of 5% CO2 for 15 days, and then they were ready to use. Each culture was genotyped (from the tail of each pup).

#### Patch-clamp recordings of cultured hippocampal neurons

Patch–clamp recordings of hippocampal neurons were carried out as previously described.^20^ Voltage clamp experiments were performed using an EPC-9 amplifier controlled by Patchmaster software (HEKA Elektronik, Lambrecht, Germany). An AgCl wire was used as a reference electrode. Solutions were applied to the cells via a homemade gravity-fed perfusion system, connected by a 5-way manifold, to a RC25 perfusion chamber (Warner Instruments, Hamden, CT, United States). The patch pipettes were pulled with a resistance of 3-5 MΩ using a DMZ-Universal Puller (Zeitz Instruments, Munich, Germany). The extracellular solution had the following composition (in mM): 140 NaCl, 5 KCl, 2 CaCl_2_, 1 MgCl_2_, 10 glucose, and 10 HEPES, pH 7.4. The pipette solution had the following composition (in mM): 140 CsCl, 10 EGTA, 0.3 Mg_2_ATP, 0.3 CaCl_2_, and 10 HEPES, pH 7.3. To prevent network activity, the experiments were performed in the presence of 1 μM tetrodotoxin (TTX), 30 μM picrotoxin, 10 μM bicuculline methiodide, 10 μM 6-cyano-7-nitroquinoxaline-2,3-dione disodium (CNQX) and 10 μM D-(-)-2-Amino-5-phosphonopentanoic acid (D-AP5) in the extracellular solution. Moreover, to prevent the activation of capacitative Ca^2+^ entry upon store depletion, the PLC inhibitor U-73122 (5 μM) was added in the extracellular medium.

#### Brain slice preparation

Mice were sacrificed by cervical dislocation, their brains were quickly collected and immersed in ice-cold artificial cerebrospinal fluid aCSF (126 mM NaCl, 3 mM KCl, 2.4 mM CaCl_2_, 1.3 mM MgCl_2_, 1.24 mM NaH_2_PO_4_, 26 mM NaHCO_3_, and 10 mM glucose). The solution was bubbled with 95%O_2_-5% CO_2_%. Brains were mounted onto vibratome and horizontal sections of 350 μm were cut in ice-cold aCSF to obtain the ventral hippocampus. Brain slices were acclimatized in oxygenated aCSF at 32°C for at least 1 h before use for the field potential recordings and for 3 hours for the slice stimulations.

#### Field potential recordings

The slices were gently transferred to a holding chamber while continuously being perfused by oxygenated aCSF (2 ml/min) at 30 °C, where they were left for 60 min to recover. Excitatory postsynaptic potentials (EPSP) were evoked through a bipolar stimulating electrode that was placed in the Schaffer collaterals and recorded by the AxoClamp 2B (Axon Instruments, Foster City, CA, USA) amplifier through a glass electrode, which was backfilled with 2M NaCl and placed in the CA1 region (stratum radiatum). The stimuli consisted of 100 μs pulses of constant currents with the intensity adjusted to produce 35% of the maximum response every min. After the placement of the electrodes, responses were stabilized for 30 to 60 min (1 stimulation/min). Responses were digitized by Digidata 1322A (Axon Instruments) and recorded to a computer using WinLTP software.^97^ Slopes of fEPSP were normalized to the baseline response defined as 100% and are presented as group means ± SEM. The input-output function was examined by stimulating slices with incrementally increasing current and recording the EPSP response. LTD was induced chemically by applying 100 μM DHPG for 5 min. All LTD experiments were done in the presence of 50 μM picrotoxin and 10 μM D-(-)-2-Amino-5-phosphonopentanoic acid (D-AP5), a potent antagonist of the N-methyl-D-aspartate receptor.

#### Acute slice stimulation

Each hippocampal region was cut out from the whole brain slice and was gently transferred to a holding chamber while continuously being perfused by oxygenated aCSF (2 ml/min) at 30 °C where they were left for 3 hours to allow recovery for protein synthesis.^103^ DHPG (100 μM) was applied for 5 min and slices were collected at different time points then snap frozen rapidly in liquid nitrogen. 2 hippocampi were collected in one Eppendorf and were considered as 1 measurement. All experiments were done in the presence of 50 μM picrotoxin and 10 μM of D-AP5.

#### Immunocytochemistry - Surface and internalized staining of AMPA receptors

Experiments were done as previously described^104^ with minor modifications. 2.5 μg (1/65 dilution) of an antibody directed at the N-terminus of the GluA1 receptor subunit, anti-GluA1 (Cell Signaling #13185) diluted in cell neuronal culture medium (Neurobasal medium supplemented with glutamine, B27, 10% FBS) was applied on live hippocampal neurons for 15 min, before being washed out with neurobasal media. Neurons were then treated (or not, as control) with 100 μM of DHPG for 5 min at 37°C, in the presence of 50 μM picrotoxin and 10 μM D-AP5, before being washed out, and cells were incubated for 60 min at 37°C to allow the endocytosis of the receptors. Cells were then incubated, on ice, with the first secondary antibody Alexa Fluor 568 (1/50) for 1 hour to label the surface GluA1 receptors. Cells were then fixed with 4% paraformaldehyde for 15 min, permeabilized with 0.2% Triton X-100 for 10 min, and blocked for 1 hour, before adding the second secondary antibody Alexa Fluor 488 (2/500) for 1 additional hour to label the internalized receptors. At the end, cells were mounted with DAPI on specific coverslips. Labeled cells were imaged using a 40X oil objective mounted on a Zeiss Axiovert. Assays, where cells were fixed without being permeabilized, have been done to determine the saturation concentration of Alexa Fluor 568 (first secondary antibody) to be used, and to make sure that Alexa Fluor 488 (second secondary antibody) only label the internalized receptors and not the surface ones. Illumination parameters were optimized based on this control experiment and subsequently kept constant across all conditions. Non-specific background was assessed through control staining in which primary antibodies were omitted. The quantitative analysis was performed using Fiji ImageJ where images background was subtracted using the process/subtract background function with the following parameters for all condition: rolling ball radius =50 pixels. The internalization index was determined by dividing the whole field fluorescence of the internalized receptors by the total fluorescence following this equation: mean intensity Alexa Fluor 488 / (mean intensity Alexa Fluor 488 + mean intensity Alexa Fluor 568). For each experimental condition, a single coverslip prepared from one mouse was analyzed. Between 10 and 20 fields were imaged per coverslip, with an average of 30 cells analyzed (n is the number of mice used in each condition).

#### Drugs

DHPG (*RS*)-3,5-Dihydroxyphenylglycine, picrotoxin, D-AP5 (D-2-amino-5-phosphonovalerate), anisomycin (2*R*,3*S*,4*S*)-2-[(4-Methoxyphenyl)methyl]-3,4-pyrrolidinediol 3-acetate), *W13 hydrochloride (N*-(4-Aminobutyl)-5-chloro-2-naphthalenesulfonamide hydrochloride), and cycloheximide (4-[2-(3,5-Dimethyl-2-oxo-cyclohexyl)-2-hydroxyethyl]-2,6-piperidinedione), U-73122 1-[6-[((17β)-3-Methoxyestra-1,3,5[10]-trien-17-yl)amino]hexyl]-1H-pyrrole-2,5-dione, were obtained from Tocris Bioscience. A-484954 (7-Amino-1-cyclopropyl-3-ethyl-1,2,3,4-tetrahydro-2,4-dioxopyrido[2,3-d] pyrimidine-6-carboxamide) was obtained from MedChemExpress. OH-tamoxifen, TTX (tetrodotoxin), bicuculline, CNQX (6-cyano-7-nitroquinoxaline-2,3-dione disodium), and U0126 (1,4-Diamino-2,3-dicyano-1,4-*bis*[2-aminophenylthio]butadiene) were obtained from Sigma. All drugs were prepared as stock solutions according to the supplier’s recommendations and stored at -20°C until use.

### Quantification and statistical analysis

Data are expressed as means ± standard error of mean (SEM). For each set of experiments, each n represents one slice (for electrophysiology), one coverslip from one animal (for immunocytochemistry), 1 hippocampal tissue or 3 CA1 regions from one animal (for RT-qPCR), 1 hippocampal tissue or 2 hippocampal slices from one animal (for western blot), 1 cell (for whole-cell patch-clamp) and one animal (for behavior testing). Graph Pad prism software was used for all statistical analysis. Statistical significance was assessed by Student’s t-test for parametric distribution and Mann-Whitney t-test for non-parametric distribution, One-way analysis of variance (ANOVA) test for multiple groups, or two-way analysis of variance (ANOVA) tests for multiple comparisons. Statistical significance was fixed to p < 0.05.
