# Supplementary material for: Conditional deletion of TRPC1 channel modulates synaptic plasticity, long term depression, and memory extinction in Fragile X syndrome mice
Source: iScience. 2025 Jul 10;28(8):113085. doi: 10.1016/j.isci.2025.113085 (PMC12329558; doi:10.1016/j.isci.2025.113085)
Supplement: Document S1. Figures S1–S6 [file mmc1.pdf]

iScience, Volume *28*

Supplemental information

Conditional deletion of TRPC1 channel modulates synaptic plasticity, long term depression, and memory extinction in Fragile X syndrome mice

Farah Issa, Xavier Yerna, Thibaud Parpaite, Caren Jabbour, Olivier Schakman, Nicolas Tajeddine, Roberta Gualdani, and Philippe Gailly

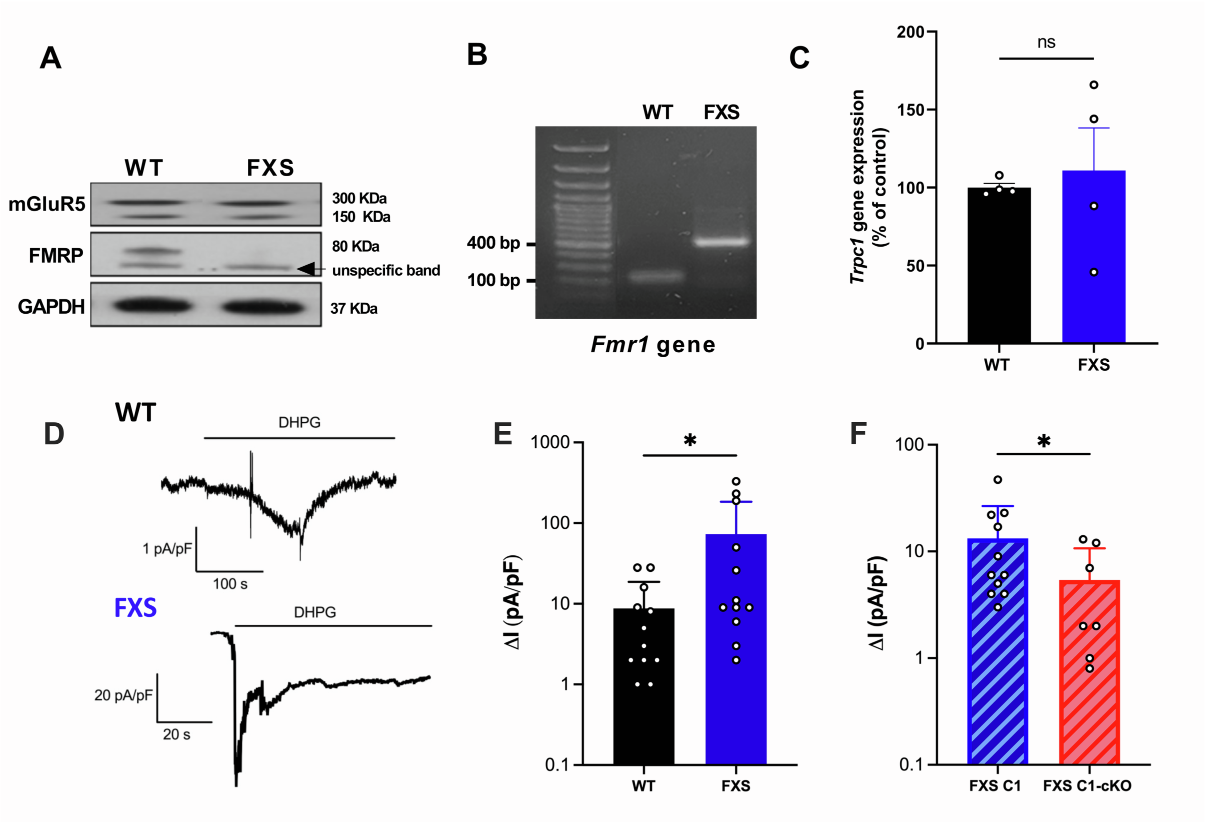

Figure S1. Characterization of FXS mice (related to Figure 1 and Methods). **(A)** Representative western blot of mGluR5 and FMRP proteins extracted from whole hippocampal tissue lysates of FXS mice and their control WT, showing the absence of the FMRP protein in FXS samples. **(B)** Representative PCR genotyping of *Fmr1* gene in WT *(~131 bp fragment)* and FXS *(~400 bp fragment)* from DNA extracts. **(C)** *Trpc1* gene expression measured by RT-qPCR analysis on whole hippocampus of WT and FXS samples showing a stable *Trpc1* expression in both groups (WT: n = 4, mean = 100% ± 2.6, FXS: n = 4, mean = 111% ± 27.1, p = 0.70, Student's t-test). ΔCT of each sample was normalized to *Gapdh*. ΔCT were then normalized to WT as control and expressed as percentage. n=*4* per group, each n represents 1 hippocampal tissue collected from 1 mouse. **(D)** Representative inward current traces recorded at –60 mV, through WT and FXS hippocampal neurons, in the presence of 100 µM DHPG, showing an increased response in FXS compared to WT. **(E)** Quantification of DHPG-induced currents in WT and FXS hippocampal neurons. Unpaired Student's t-test done after logarithmic transformation of the data to normalize variances. *: p = 0.023, n = 12 WT and 12 FXS patched cells from 4 different cultures. **(F)** Quantification of inward current traces recorded at –60 mV, through FXS C1 and FXS C1-cKO hippocampal neurons treated one week with hydroxytamoxifen, in the presence of 100 µM DHPG, showing a decreased response after TRPC1 knock down. Unpaired Student's t-test done after logarithmic transformation of the data to normalize variances. *: p = 0.04, n = 11 FXS C1 and 7 FXS C1-cKO patched cells, from 2 different cultures.

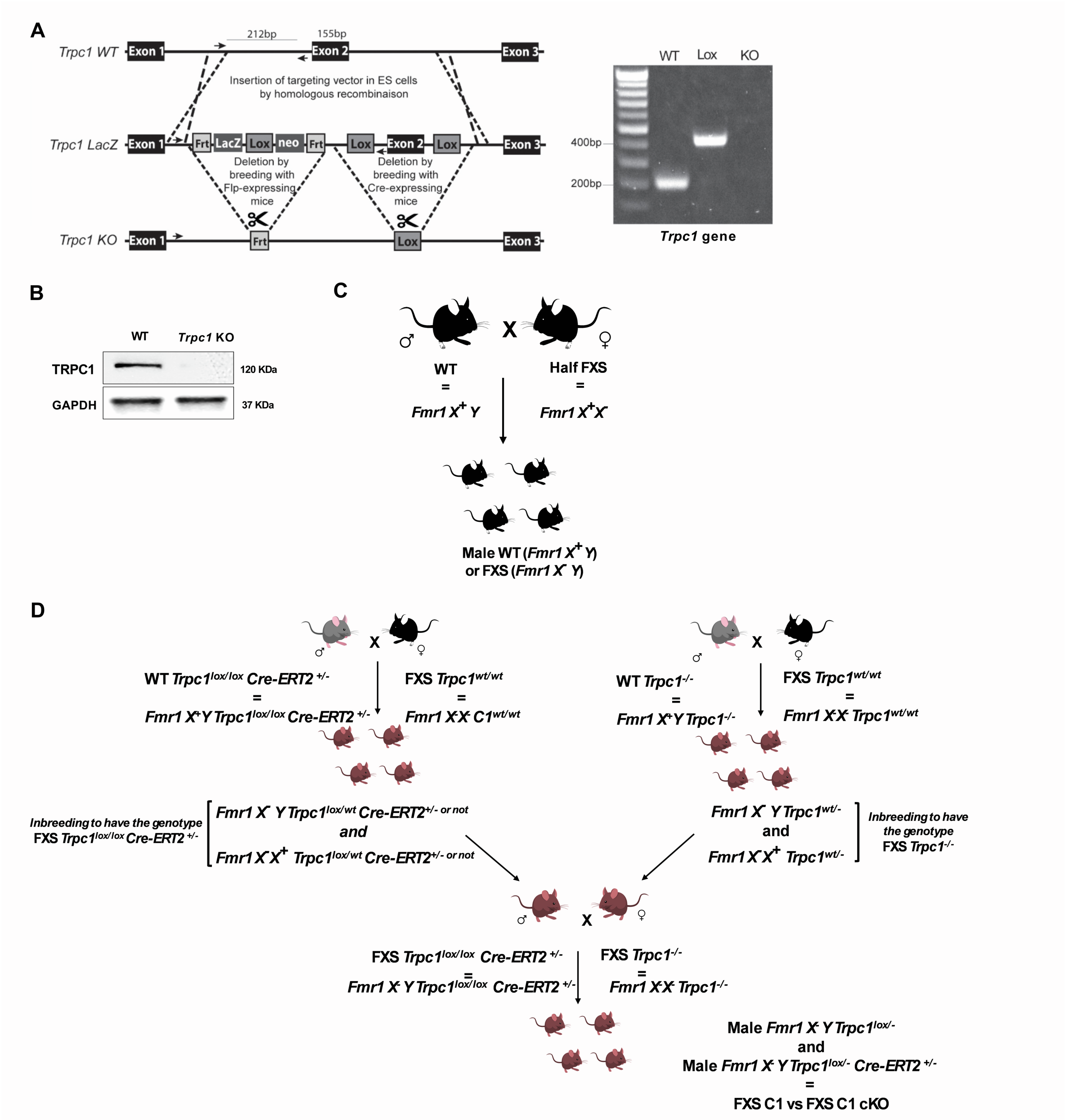

Figure S2. Generation and characterization of FXS C1-cKO mice (related to Methods). (A) Adapted figure from our previous work (Lepannetier et al., 2018) showing construction schematic of Trpc1-LacZ reporter and Trpc1 KO alleles, and PCR products. Sketch 1 depicts the WT locus. Regions of homologous recombination are indicated by crossover lines, while arrows mark the sequences targeted by PCR primers. Sketch 2 illustrates the Trpc1 gene integrated with the targeting vector, which includes a LacZ reporter cassette flanked by FRT recombination sites and exon 2 flanked by loxP recombination sites. Sketch 3 shows the modified Trpc1 locus following FLP-mediated removal of LacZ and the disrupted gene structure after Cre-mediated recombination. PCR gel at the right represents genotyping of Trpc1$^{wt/wt}$ (212 bp fragment), Trpc1$^{lox/lox}$ (inserted lox sequence: 420 bp fragment), and Trpc1$^{-/-}$ (KO allele where the reverse primer site is not present). (B) Representative western blot for TRPC1 antibody validation using Trpc1 KO hippocampal tissues vs WT hippocampal tissues. (C) Mating scheme to generate littermates of male FXS and WT mice. B6.129P2-Fmr1tm1Cgr/J male mice were crossed with their control WT C57BL/6J female mice to produce half FXS female mice Fmr1 X^{+}X^{-}. These female mice were then crossed further with their male control WT C57BL/6J mice to produce littermates of WT and FXS genotypes. (D) Mating scheme to generate littermates of male FXS C1 (Fmr1 X^{-}Y Trpc1$^{lox/-}$) and FXS C1-cKO (Fmr1 X^{-}Y Trpc1$^{lox/-}$ Cre-ERT2$^{+/-}$). WT Trpc1$^{lox/lox}$ Cre$^{+/-}$ were crossed with FXS Trpc1$^{wt/wt}$ and littermates were then further intercrossed to generate FXS Trpc1$^{lox/lox}$ Cre-ERT2$^{+/-}$. In parallel, WT Trpc1$^{-/-}$ were crossed with FXS Trpc1$^{wt/wt}$ and littermates were then further intercrossed to generate FXS Trpc1$^{-/-}$. FXS Trpc1$^{lox/lox}$ Cre-ERT2$^{+/-}$ were crossed with FXS Trpc1$^{-/-}$ to generate in the end FXS C1 vs FXS C1-cKO. Only male mice were used for all the experiments.

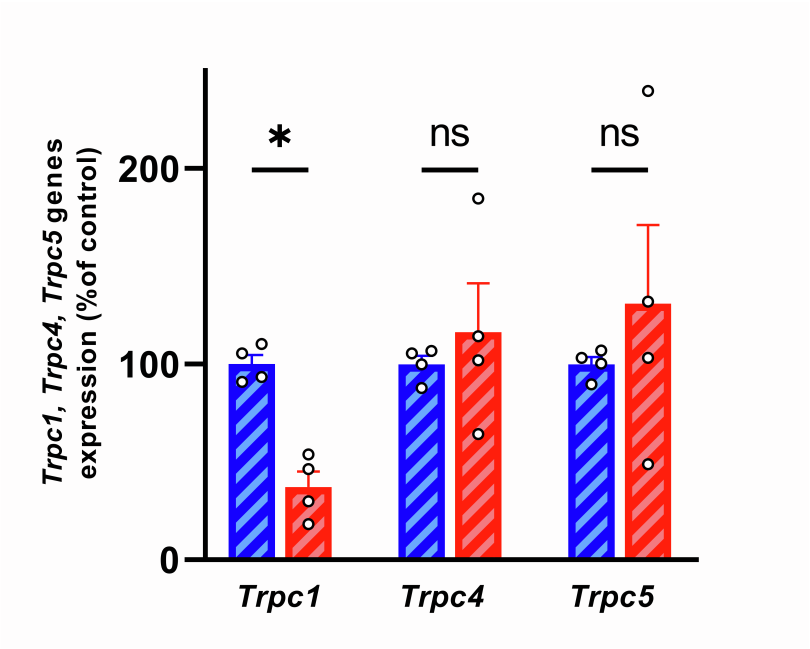

Figure S3. *Trpc1*, *Trpc4*, and *Trpc5* genes expression (related to Figure 1). *Trpc1*, *Trpc4*, and *Trpc5* gene expression measured by RT-qPCR analysis on the CA1 region of the hippocampus of FXS C1 and FXS C1-cKO 10 days post tamoxifen injection, showing a decrease in *Trpc1* gene expression (FXS C1: mean = 100% ± 4.6, FXS C1-cKO: mean = 37.1% ± 8, $p = 0.016$) in the FXS C1-cKO compared to their control FXS C1, and an unchanged *Trpc4* (FXS C1: mean = 100% ± 4.3, FXS C1-cKO: mean = 116.3% ± 25.1, $p = 0.50$) and *Trpc5* (FXS C1: mean = 100% ± 3.7, FXS C1-cKO: mean = 131% ± 40.1, $p = 0.21$) genes expression between the two groups. ⊿CT of each sample was normalized to *Gapdh*. ⊿CT were then normalized to FXS C1 as control and expressed as percentage ($n = 4$ for each group, each n represents 3 CA1 regions collected from 1 mouse, One-way ANOVA). * $p < 0.05$, ns not significant.

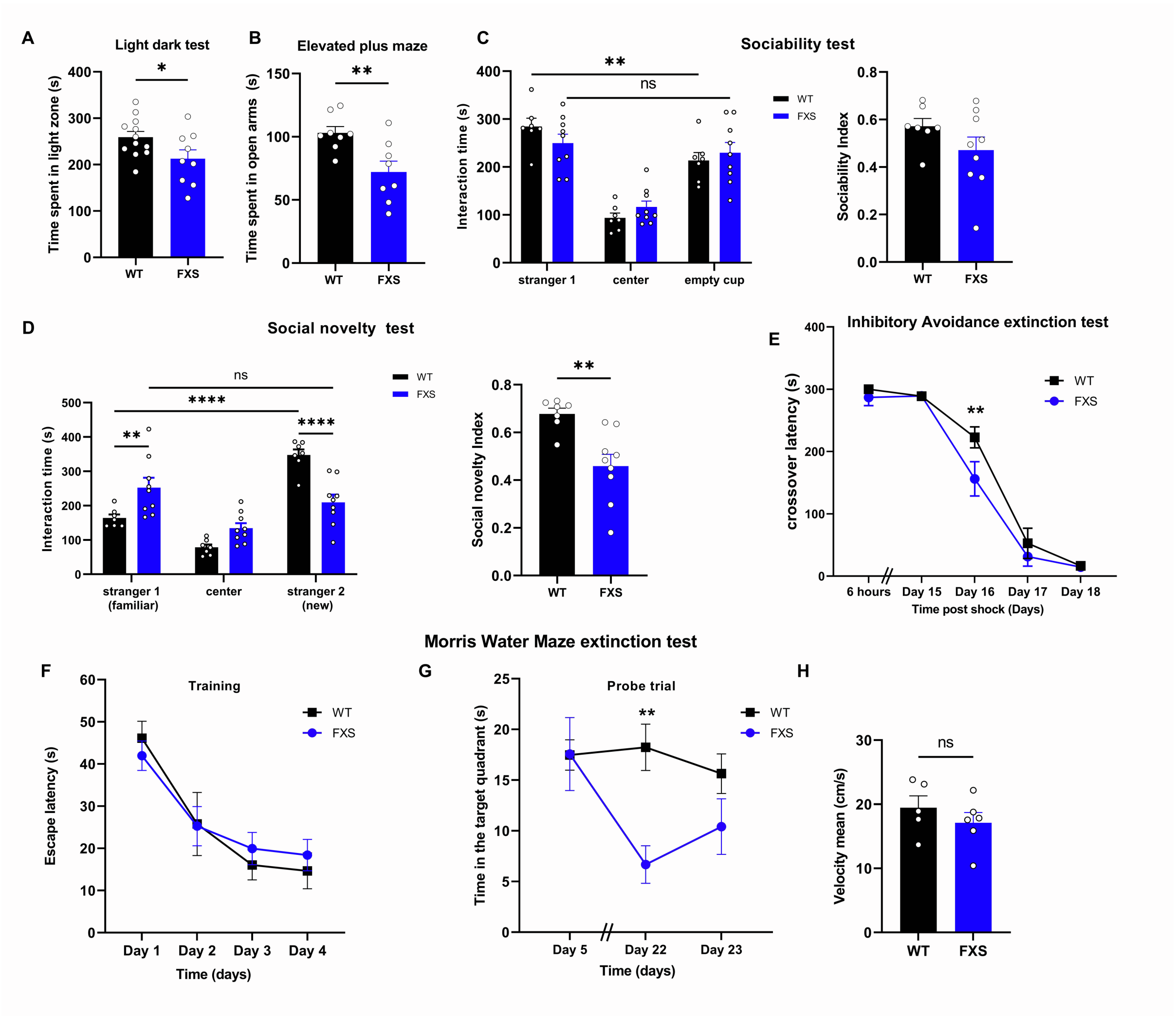

Figure S4. Behavioral evaluation of anxiety, sociability, and memory extinction in FXS and WT mice (related to Figure 2). (A,B) Light dark test (LDT), and Elevated plus maze (EPM) assess anxiety behavior in FXS and WT groups. Time spent in the light zone and time spent in open arms were lower in the FXS group compared to their control WT in LDT (WT n=12 mean = 259.2 ± 12.3, FXS: n=9 mean = 212.8 ± 18.9, Student's t-test, p = 0.044), and EPM (WT n=8, mean = 103.1 ± 5.1, FXS: n = 8, mean = 72.03 ± 8.6, Student's t-test, p = 0.007). **(C,D)** Sociability and Social novelty tests in FXS and WT groups. FXS presented no preference in the sociability test (sociability index: WT: n = 7, mean = 0.57 ± 0.03, FXS: n = 9, mean = 0.47 ± 0.05, Student's t-test, p = 0.17) but a reduced attraction in the social novelty test (social novelty index: WT: n = 7, mean = 0.67 ± 0.02, FXS: n = 9, mean = 0.45 ± 0.04, Student's t-test, p = 0.002). **(E)** Inhibitory avoidance (IA) extinction test where crossover latency was assessed for FXS vs WT, 6 hours, and 15 to 18 days post-shock. FXS showed an enhanced memory extinction compared to WT on day 16 of the test (WT n=10, mean = 222.7 ± 16.8, FXS: n = 7, mean = 156.1 ± 27.5, Two-way ANOVA, p = 0.009). **(F – H)** Morris Water Maze (MWM) extinction test for FXS vs WT, showing escape latency, time in target quadrant, and velocity mean (on day 5 of the test) respectively. FXS presented a normal initial reference memory (probe trial on day 5, WT: n = 5, mean = 17.48 ± 1.4, FXS: n = 6, mean = 17.56 ± 3.5, Two-way ANOVA, p = 0.982) but an enhanced memory extinction in the repeated probe trials (WT: n =5, mean = 18.2 ± 2.2, FXS: n = 6, mean = 6.6 ± 1.8, Two-way ANOVA, p = 0.003 on day 22, and WT: mean = 15.6 ± 1.8, FXS: mean = 10.4 ± 2.7, Two-way ANOVA , p = 0.161 on day 23). Values are means ± SEM. * p < 0.05, ** p < 0.01, **** p < 0.0001, ns not significant.

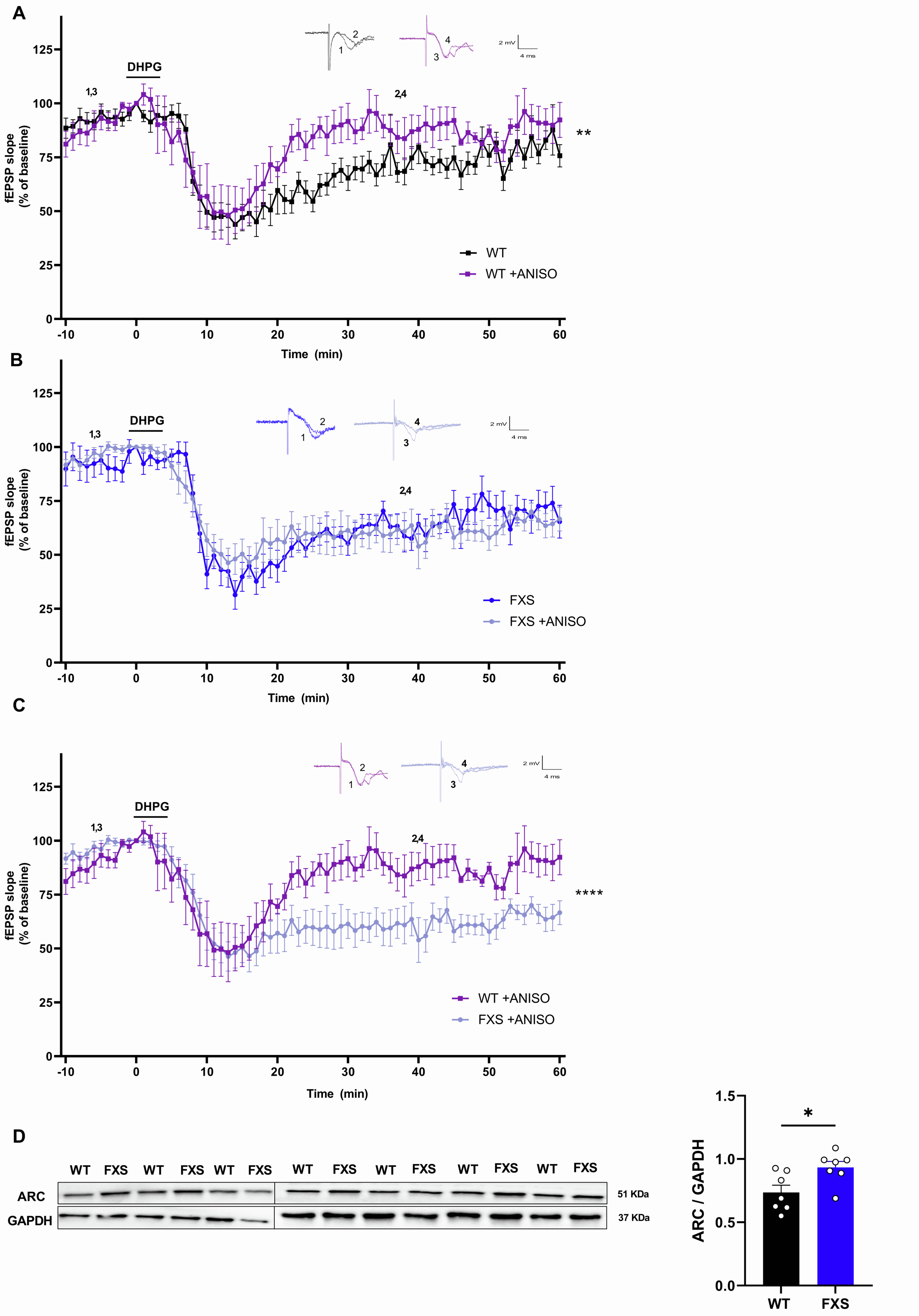
A
B
C
D
DHPG
1,3
2,4
1
2
3
4
2 mV
4 ms
fEPSP slope
(% of baseline)
125
100
75
50
25
0
-10
0
10
20
30
40
50
60
Time (min)
**
****
WT
WT +ANISO
FXS
FXS +ANISO
WT
FXS
ARC
GAPDH
51 KDa
37 KDa
ARC / GAPDH
1.5
1.0
0.5
0.0
*

Figure S5. Characterization of DHPG-induced LTD in hippocampal SC-CA1 in FXS and WT mice (related to Figure 3). (A) Time-course of field excitatory postsynaptic potentials (fEPSP) slopes measured in CA1 stratum radiatum, before and after stimulation with 100 µM DHPG in slices from WT and WT in the presence of 20 µM anisomycin (WT +ANISO) 30 min before-DHPG. Upper insets show representative traces of fEPSP before and after DHPG. (WT n=14, WT +ANISO n=6, n representing the number of slices from 5 to 9 different mice in each group, Two-way ANOVA, p (time x genotype) = 0.0049. **(B)** Time-course of field excitatory postsynaptic potentials (fEPSP) slopes measured in CA1 stratum radiatum, before and after stimulation with 100 µM DHPG in slices from FXS and FXS in the presence of 20 µM anisomycin (FXS +ANISO) 30 min before-DHPG. Upper insets show representative traces of fEPSP before and after DHPG (FXS n=9, FXS +ANISO n=8, n representing the number of slices from 5 to 9 different mice in each group, Two-way ANOVA, not significant. **(C)** Time-course of field excitatory postsynaptic potentials (fEPSP) slopes measured in CA1 stratum radiatum, before and after stimulation with 100 µM DHPG in slices from WT +ANISO and FXS+ANISO. Upper insets show representative traces of fEPSP before and after DHPG. (WT +ANISO n=6, FXS +ANISO n=8, n representing the number of slices from 5 to 9 different mice in each group, Two-way ANOVA, p (time x genotype) < 0.0001. **(D)** Representation and quantification of western blots for ARC protein expression normalized to GAPDH, in hippocampal tissues in WT and FXS (WT n=7, mean = 0.73 ± 0.05, FXS: n=7, mean = 0.93 ± 0.04, each n represents 1 hippocampal tissue collected from 1 mouse, Student's t-test, p = 0.028). Values are means ± SEM. * p < 0.05, ** p < 0,01, **** p < 0.0001.

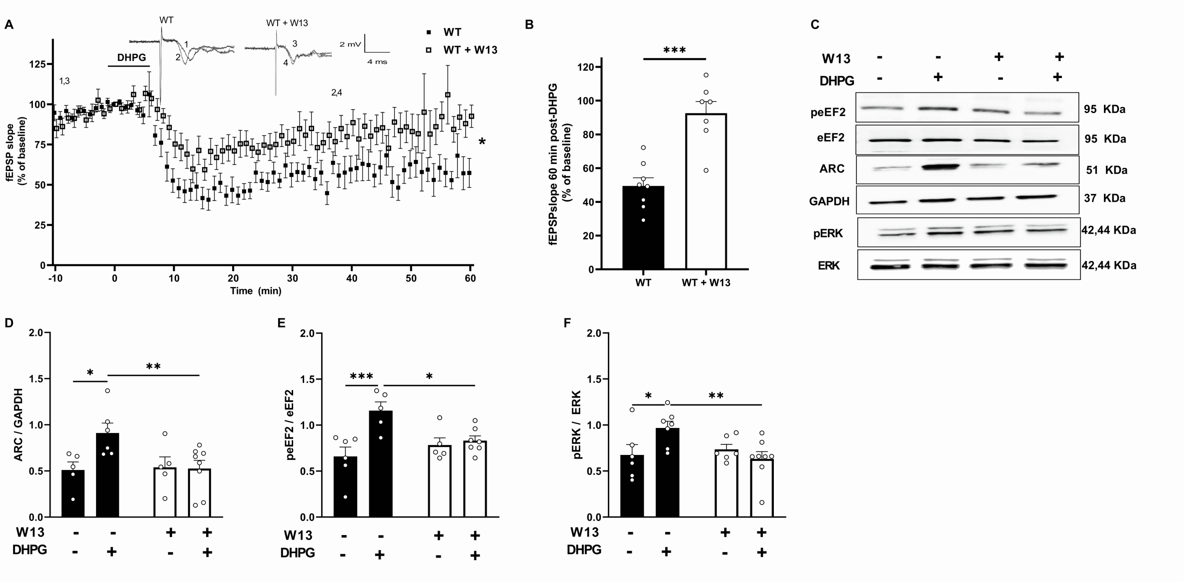

Figure S6. W13 hydrochloride (calmodulin antagonist) effects on DHPG-induced LTD and on protein expressions in hippocampal WT mice (related to Figure 7). A, Time-course of field excitatory postsynaptic potentials (fEPSP) slopes measured in CA1, before and after stimulation with 100 µM DHPG in slices from WT and WT +W13 (70 µM W13, 30 min before-DHPG). Upper inserts show representative traces of fEPSP before and after DHPG. (WT n=8, WT +W13 n=7, n representing the number of slices from 5 different mice in each group, Two-way ANOVA, F (1, 13) = 6.441, p=0.0247, genotype effect) **B,** Quantification of the averaged fEPSP slope 60 min post-DHPG induction in both groups, showing a maintained LTD in WT, but not in WT +W13. (WT: n=8, mean = 49.4% ± 4.8, WT +W13: n = 7, mean = 92.5% ± 6.9, Student's t-test, p = 0.0002), n representing the number of slices from 5 different mice in each group. **C - F,** Representation and quantification of western blots for ARC, peEF2, and pERK proteins in hippocampal slices stimulated with 100 µM DHPG in WT and WT +W13, slices were collected 0 min and 5 min after DHPG stimulation (70 µM W13, 30 min before-DHPG). (ARC expression at 5 min: WT: n = 6, mean = 0.91 ± 0.1, WT +W13: n = 8, mean = 0.52 ± 0.08, Two-way ANOVA, p = 0.008, peEF2 expression at 5 min: WT: n = 5, mean = 1.15 ± 0.09, WT +W13: n = 7, mean = 0.83 ± 0.05, Two-way ANOVA, p = 0.010, and pERK expression at 5 min: WT: n =7, mean = 0.96 ± 0.07, WT +W13: n = 8, mean = 0.63 ± 0.07, Two-way ANOVA, p = 0.005), n representing the number of hippocampal slices collected from 4 to 6 different mice in each group. All experiments were done in the presence of 50 µM picrotoxin and 10µM D-AP5. Values are means ±SEM. * p<0.05, ** p < 0.01, *** p<0.001.
